# Injectable HAMA-CPC hydrogels loaded with high-yield 3D bioprinted adipose-derived stem cell small extracellular vesicles for increased bone repair

**DOI:** 10.1186/s12951-025-03596-4

**Published:** 2025-07-21

**Authors:** Wenbin Xu, Wenling Gao, Yi Zhang, Gang Hou, Wenhui Zhang, Jintao Deng, Kun Wang, Yichun Xu, Boxun Liu, Tao Xu, Chang Liu, Tangzhao Liang

**Affiliations:** 1 Department of Orthopaedic Surgery, The Third Affiliated Hospital of Sun Yat-sen University, Guangzhou, 510630 China; 2 Department of Orthodontics, Hospital of Stomatology, Sun Yat-sen University, Guangzhou, 510060 China; 3 Research and Development Department, Huamei Biotech Co. Ltd, Shenzhen, 518107 China; 4 Department of Joint and Trauma Surgery, The Third Affiliated Hospital of Sun Yat-sen University, Guangzhou, 510630 China; 5 Center for Bio-Intelligent Manufacturing and Living Matter Bioprinting, Research Institute of Tsinghua University in Shenzhen, Shenzhen, 518057 China; 6 Tsinghua Shenzhen International Graduate School, Tsinghua University, Shenzhen, 518055 China

**Keywords:** 3D cultured adipose-derived stem cell small extracellular vesicles, Bone repair, Angiogenesis, Hyaluronic acid methacrylate (HAMA), Calcium phosphate cement (CPC)

## Abstract

In the field of bone tissue engineering, the development of effective strategies for bone defect repair remains a major challenge. Herein, we report a novel approach involving the integration of high-yield 3D bioprinted adipose-derived stem cell small extracellular vesicles (3D-sEVs) into an injectable HAMA-CPC hydrogel (HAMA-CPC@3D-sEVs). In vitro, HAMA-CPC@3D-sEVs significantly promoted the proliferation, migration, and osteogenic differentiation of bone marrow stromal cells (BMSCs) and promoted angiogenesis in human umbilical vein endothelial cells (HUVECs). In vivo, in a rat tibial defect model, this treatment strongly promoted bone regeneration, increasing the bone volume fraction (BV/TV), bone volume (BV), and trabecular thickness (Tb.Th) at 4 weeks post-surgery. Mechanistically, proteomic analysis revealed that NAMPT in 3D-sEVs upregulated S1PR1 in HUVECs, leading to increased VEGF expression and angiogenesis. These findings suggest that 3D-sEVs-functionalized HAMA-CPC hydrogels have good potential for bone defect repair.

**Graphical abstract:**

**Figure:**
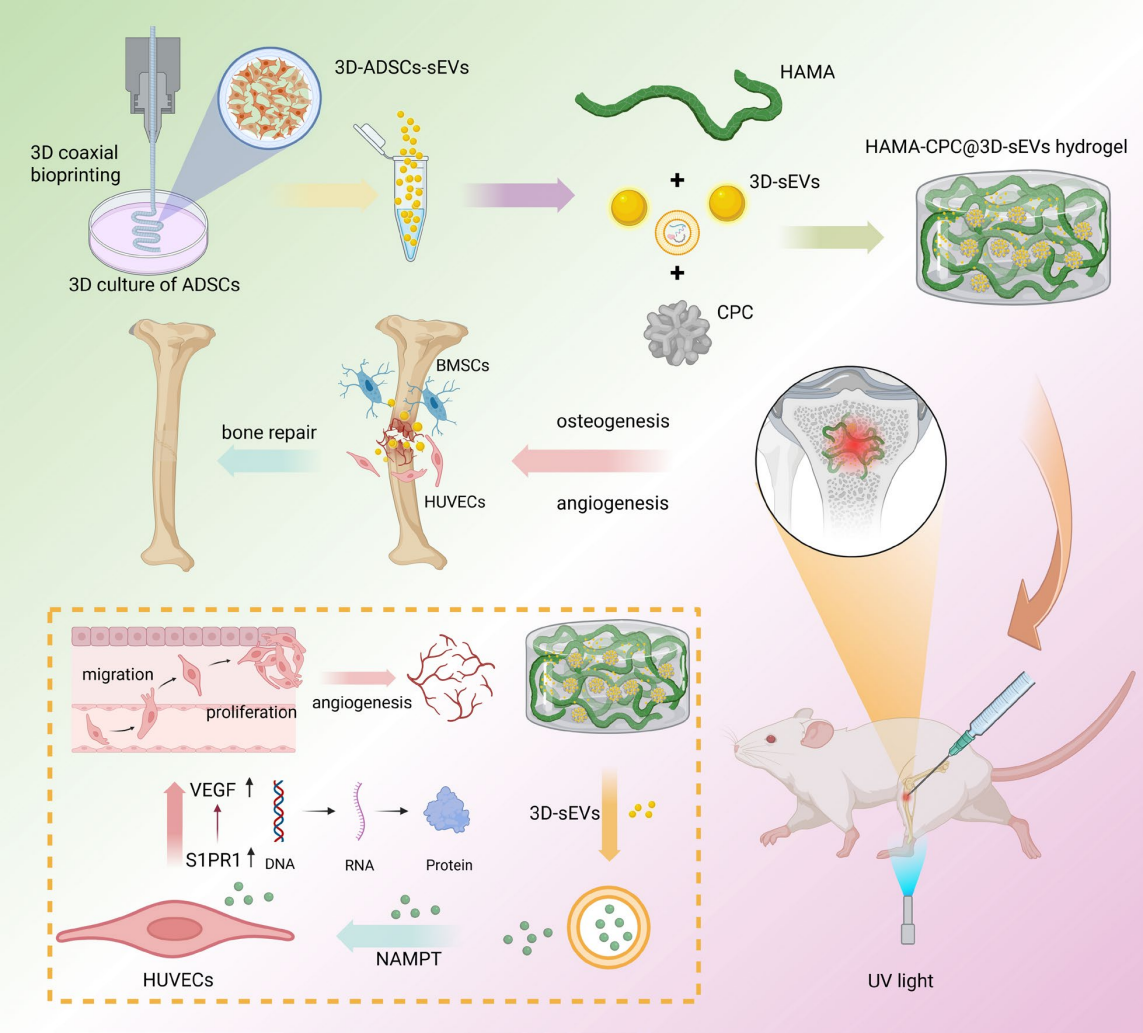
Schematic diagram of the overall study design of HAMA-CPC@3D-sEVs for the bone regeneration in rat

**Supplementary Information:**

The online version contains supplementary material available at 10.1186/s12951-025-03596-4.

## Introduction

Bone defects, arising from trauma, tumors, congenital disorders, and infectious diseases, pose major challenges in orthopedic surgery [1, 2]. Standard treatment options such as autografts, allografts, and synthetic grafts have limitations [3, 4]. Despite their immunocompatibility, autografts are hindered by donor site morbidity and limited bone availability [5, 6]. Allografts face immune rejection and suboptimal biocompatibility [7], whereas synthetic grafts have issues with integration and potential rejection [8, 9]. Thus, developing effective bone substitutes is crucial in bone tissue engineering, necessitating innovative approaches.

Mesenchymal stem cells (MSCs) are important for bone repair because of their pluripotency [10]. However, MSC-based therapies face obstacles such as donor site morbidity and concerns about immune rejection and uncontrolled cell proliferation [11]. Notably, recent scientific breakthroughs have uncovered the substantial therapeutic potential of small extracellular vesicles (sEVs), also known as exosomes, derived from MSCs. To maintain scientific rigor and align with the MISEV2023 guidelines, we consistently use the term “sEVs” throughout this study [12]. These nanoscale vesicles encapsulate many bioactive molecules that are capable of emulating the regenerative capabilities of MSCs while circumventing the risks inherent to cell transplantation [11, 13]. MSC-derived sEVs play pivotal roles in mediating intercellular communication, thereby influencing essential physiological processes such as cell proliferation, the modulation of inflammatory responses, angiogenesis, and immune regulation [14, 15]. Owing to their inherent stability, convenient storage properties, and ability to permeate biological barriers, these materials are highly scalable and a promising alternative for applications in regenerative medicine and tissue engineering [16, 17]. This emerging paradigm shift shows promise for addressing current limitations and advancing the field toward more effective and safer bone defect repair strategies.

The traditional two-dimensional (2D) cell culture modalities employed for sEVs generation fail to recapitulate the intricate in vivo milieu, thereby negatively affecting cellular morphology, viability, stemness attributes, and differentiation potential [18, 19]. In response to these constraints, three-dimensional (3D) culture techniques have emerged as a viable alternative, providing augmented sEVs characteristics [20]. Compared with their 2D counterparts, sEVs derived from 3D cultures more faithfully mimic natural cellular behaviors, augment cell-to-cell and cell-to-matrix interactions, display augmented biological activity, and yield substantially greater quantities [21, 22]. Coaxial bioprinting, an advanced extrusion-based 3D bioprinting methodology, enables the fabrication of high-density cell constructs, exemplified by vascular, tumor, and neural models [23, 24]. Through encapsulation of cells within hollow microfibers, this technique not only facilitates cell migration and interaction but also remodels the microenvironment [25]. Our research group has successfully developed a robust 3D cell culture platform leveraging coaxial bioprinting, which has substantially increased the efficacy of sEVs production [26]. The incorporation of the sEVs obtained through this approach into a PCL-GelMA scaffold was shown to accelerate skin wound healing, manifested by increased collagen deposition, epithelial regeneration, and angiogenesis [27]. However, the precise mechanisms and impacts of coaxial 3D bioprinting-derived sEVs on bone defect repair remain unclear and warrant further in-depth exploration.

sEVs have emerged as highly promising candidates for bone defect repair owing to their good capacity to stimulate tissue regenerative processes. However, their in vivo application is impeded by rapid clearance, which negatively affects their long-term therapeutic efficacy [28]. Injectable hydrogels offer a potential solution by serving as carriers for sEVs, enabling their sustained release and thereby prolonging the period during which they can exert their beneficial effects, a crucial aspect considering the protracted time frame typically required for effective bone healing [14, 29]. Hyaluronic acid methacrylate (HAMA) has been widely recognized for its favorable injectability and excellent biocompatibility [30, 31], making it an attractive component in the context of tissue engineering. In the context of bone regeneration, HAMA can adapt well to irregular defect geometries, facilitating the filling of complex voids [30, 32]. However, notably, HAMA alone may lack sufficient osteogenic inductive properties. In contrast, calcium phosphate cement (CPC) is renowned for its outstanding osteoconductive capabilities, providing a favorable microenvironment for bone cell attachment, proliferation, and differentiation [9, 33]. Nevertheless, CPC has drawbacks like imperfect injectability, slow solidification, and slow degradation with long-term impacts [34, 35]. The combination of HAMA and CPC into a composite hydrogel is hypothesized to overcome the individual limitations of each [36]. This proposed composite is expected to increase sEVs stability and retention and synergistically integrate HAMA adaptability with the bone-regenerative traits of CPC, potentially creating an optimal sEVs delivery vehicle for bone defect repair. This system is also anticipated to mimic the native bone matrix and offer tunable properties for customization, increasing its clinical potential.

In this study, we employed coaxial bioprinting to encapsulate adipose-derived stem cells (ADSCs) at a high density within hydrogel fibers, which enabled the effective generation of 3D-cultured ADSC-derived sEVs (3D-sEVs). These sEVs were subsequently integrated into a novel composite hydrogel composed of HAMA and CPC (HAMA-CPC). We conducted in vitro investigations into the osteogenic and angiogenic capabilities of HAMA-CPC@3D-sEVs and further explored their role in promoting tibial defect repair in rats through animal experiments. The overarching aim of this research was not only to validate the regenerative potential of 3D-sEVs but also to establish a novel and efficacious therapeutic strategy for bone defect repair, thereby contributing to the advancement of regenerative medicine in the field of orthopedics.

## Materials and methods

### Isolation and characterization of ADSC-sEVs

Human ADSCs (hADSCs) from Sciencell™ Research Laboratories were cultured in 150-mm dishes at 37 °C with 5% CO₂ in Dulbecco’s modified Eagle’s medium (DMEM) (Gibco, USA) supplemented with 10% fetal bovine serum (FBS) (Gibco, USA) and 1% penicillin‒streptomycin (Gibco, USA), and serum-free MSC chemically defined medium (Sciencell, USA) was used for sEVs collection. Coaxial 3D cell microfibers were fabricated following a previous method with a double-layer coaxial needle (inner diameter: 0.38 mm, filled with 3 × 10^8^ cells/mL ADSC suspension in defined medium; outer diameter: 1.1 mm, filled with 1.5% sodium alginate). The extrusion rates were controlled by a syringe pump, with the inner materials extruded at 3-10 ml/h and the outer at 15-30 ml/h. Gelation took place in 3% CaCl₂, followed by rinsing and culturing [26]. For 2D culture, the cells were seeded and washed with PBS, and conditioned medium was collected after 48 h; for 3D culture, the conditioned medium was mainly collected every 24 h, as our previous research indicated that this interval could obtain more EVs compared to 48-hour collection [26]. sEVs were isolated from the conditioned medium of 2D and 3D cultures via differential ultracentrifugation with an Optima MAX-XP ultracentrifuge (Beckman Coulter, USA). sEVs morphology and ultrastructure were analyzed via transmission electron microscopy (TEM) (JEOL, Japan), and the yield was quantified by a microBCA protein assay kit (CWBio, China) and nanoparticle tracking analysis (NTA). Western blotting was used to identify markers [CD63, CD81, and TSG101 (Abcam, USA)] on the sEVs.

### Materials preparation and characterization

#### Preparation of HAMA-CPC composite hydrogels

HAMA was purchased from EFL (EngForLife, China) and prepared following the manufacturer’s instructions. First, 10 mL of PBS was added to a brown bottle containing the initiator LAP (0.025 g LAP). Then, 1 g of HAMA (HAMA-150 K) was mixed into the LAP solution and stirred at room temperature for 1 h until the solution became clear and no visible particles remained. The solution was subsequently sterilized via a 0.22-µm sterile filter.

The CPC used in this study was a commercial self-setting calcium phosphate artificial bone (Shanghai Rebone Biomaterials Co., Ltd., China) consisting of calcium phosphate salts (powder) and a setting solution (soluble aqueous phosphate solution). The powder and setting solution were thoroughly mixed to form a CPC paste. This CPC paste was then added to the HAMA solution to prepare composite hydrogels with CPC concentrations of 5% (w/v), 10% (w/v), and 20% (w/v), designated HAMA-5CPC, HAMA-10CPC, and HAMA-20CPC, respectively. The mixtures were stirred magnetically for 1 h at 37 °C in a water bath while being kept away from light to prevent premature photocrosslinking. The hydrogels were photocrosslinked under 405 nm light for 10–20 s.

#### Chemical and physical characterization of hydrogels

The chemical properties of HAMA, CPC, HAMA-5CPC, HAMA-10CPC, and HAMA-20CPC were characterized via Fourier transform infrared spectroscopy (FTIR, Thermo Fisher, USA). The morphologies of HAMA and HAMA-CPC were examined by scanning electron microscopy (SEM, Carl Zeiss, Germany). Rheological properties of the composite hydrogels were evaluated using a rheometer (HAAKE MARS IQ Air, Thermo Scientific, US) equipped with a 20 mm diameter plate and a 0.3 mm gap. Oscillatory frequency sweep was conducted at a constant strain of 0.1–1000% within 0.1–100 rad/s at room temperature to determine G’ and G’’. Strain amplitude sweep was carried out at room temperature with 1 Hz rad/s and 1 − 1000% strain to detect the critical strain point. Temperature sweep was performed at a constant shear rate of 1% strain and 1 Hz within 10 °C − 45 °C to study temperature effect on viscosity. Viscosity as a function of shear rate was measured at room temperature within 0.1–1000 s⁻¹. Before each test, samples were equilibrated at room temperature for 10 min, and all measurements were done in triplicate.

#### Fabrication of sEVs-loaded HAMA-CPC hydrogels

For the preparation of sEVs-loaded hydrogels, 5 mL of PBS containing 2D-sEVs or 3D-sEVs was slowly added dropwise to the previously prepared HAMA solution. Subsequently, the CPC paste corresponding to the selected 10% (w/v) concentration was added to the HAMA-sEVs mixture. The resultant mixture was denoted as HAMA-CPC@2D-sEVs when 2D-sEVs were used, and HAMA-CPC@3D-sEVs when 3D-sEVs were used. The mixture was then magnetically stirred at 37 °C in the dark for 1 h to avoid premature photocrosslinking, followed by photocrosslinking under 405 nm light for 10–20 s.

#### In vitro release assay of sEVs from hydrogels

The release rate of sEVs from the hydrogel was determined via the BCA method. In brief, hydrogels loaded with sEVs at a concentration of 1 µg/µL were placed in PBS at 37 °C. The supernatant was collected every 2 days for 16 consecutive days, and the protein concentration was determined via a BCA assay kit. The number of released sEVs was calculated, and a release curve was plotted.

To address potential interference from hydrogel-derived proteins in the BCA results and specifically quantify sEVs, we additionally performed a CD63-specific ELISA. Using a commercial kit (Abcam, USA), supernatants collected during the incubation were analyzed following the manufacturer’s protocol. Plate wells were coated with a CD63 capture antibody, and samples were incubated for 2 h at room temperature after washing. A peroxidase-conjugated detection antibody was added post-wash and incubated for 1 h, followed by substrate addition and absorbance measurement at 450 nm using a microplate reader. Purified sEVs were used to generate a standard curve for determining sample concentrations, enabling the plotting of a specific sEVs release curve to validate the BCA data.

#### Preparation of extract liquids

The hydrogels were sterilized under UV light for 30 min, then cut into uniform fragments (2 × 2 × 2 mm) using a sterile mold. The fragments were incubated in DMEM/F12 medium (Gibco, USA) supplemented with 0.1% bovine serum albumin (BSA, Sigma), 1% penicillin/streptomycin, and 1× protease/phosphatase inhibitor cocktail (Thermo Fisher) at a material-to-medium ratio of 0.1 g/mL. The samples were gently agitated at 4 °C for 24 h to allow dissolution and release of soluble factors while minimizing sEVs degradation. The resultant extract was centrifuged at 300×g for 10 min to remove large debris, then filtered through a 0.22-µm PVDF low-protein-binding membrane. The filtrate was aliquoted and stored at -80 °C; thawed extracts were used within 2 h after ice-bath thawing.

### In vitro cell experiments

#### sEVs internalization assay

2D-sEVs and 3D-sEVs were labeled with PKH67 (Sigma, USA) following the manufacturer’s instructions. Excess dye was removed via 0.5% BSA/PBS and centrifugation. Both cell types were seeded in confocal dishes and treated with labeled sEVs for 24 h. After incubation, the cells were washed with PBS and fixed with 4% paraformaldehyde. The cytoskeleton and nuclei were stained with rhodamine (Solarbio, China) and DAPI (Thermo Fisher, USA). The uptake of 2D-sEVs and 3D-sEVs was observed via confocal laser scanning microscopy (Carl Zeiss, Germany).

#### Cell proliferation and live/dead assays

Bone marrow stromal cells (BMSCs) (Solarbio, China) or human umbilical vein endothelial cells (HUVECs) (Gibco, USA) were seeded at a density of 1 × 10⁴ cells/well in 96-well plates and cultured in the extract liquids of HAMA-CPC, HAMA-CPC@2D-sEVs, or HAMA-CPC@3D-sEVs for 1, 3, or 5 days. Cell proliferation was assessed via the Cell Counting Kit-8 (CCK-8) assay (Sigma, USA). After the samples were washed with PBS, 10% (v/v) CCK-8 solution was added, and the samples were incubated in the dark for 1 h. Optical density (OD) values were measured at 450 nm via a microplate reader (BiotekEpoch, USA).

For the live/dead assay, HUVECs or BMSCs were seeded at a density of 3 × 10⁴ cells/well in 24-well plates and cultured in the extract liquids of HAMA or HAMA-CPC for 24 h, with a control group. After that, the cells were stained via a live/dead staining kit (Thermo Fisher, USA) according to the manufacturer’s instructions. Fluorescence images were captured with an inverted fluorescence microscope (Carl Zeiss, Germany) and analyzed via ImageJ software.

For the EdU assay, HUVECs or BMSCs were divided into three groups: Ctrl group, HAMA extract-treated group, and HAMA-CPC extract-treated group. Cells in each group were seeded in 24-well plates until 70–80% confluence. EdU (Thermo Fisher, USA) was added at a final concentration of 10 µM, and the cells were incubated at 37 °C for 2 h. After being washed with PBS, the cells were fixed with 4% paraformaldehyde, permeabilized with 0.5% Triton X-100, and stained with the Click reaction mixture. Nuclei were counterstained with DAPI, and images were captured via an inverted fluorescence microscope (Carl Zeiss, Germany).

#### Cell migration assays

For evaluation of cell migration, wound healing and Transwell assays were performed. For the wound healing assay, BMSCs or HUVECs were seeded in 6-well plates at 5 × 10⁵ cells/well. After reaching 90% confluence, a scratch was made with a pipette tip, and the cells were treated with the extract liquids of HAMA-CPC, HAMA-CPC@2D-sEVs, or HAMA-CPC@3D-sEVs. Images were taken at 0 and 24 h, and the migration area was analyzed via ImageJ software.

For the Transwell assay, 100 µL of cell suspension (1 × 10⁴ cells/mL) was added to the upper chamber of a Transwell insert. The lower chamber contained medium supplemented with the extract liquids of HAMA-CPC, HAMA-CPC@2D-sEVs, or HAMA-CPC@3D-sEVs. After 12 h, the migrated cells were fixed, stained with crystal violet, and counted under a microscope.

#### Osteogenic differentiation of BMSCs

BMSCs were cultured in DMEM supplemented with 10% FBS and 1% penicillin-streptomycin. For osteogenic differentiation, BMSCs were seeded in 12-well plates at a density of 1 × 10⁵ cells/well and cultured in an osteogenic medium (OM) (Hysigen, China). For evaluation of the osteogenic effects of different hydrogels or hydrogel-sEVs complexes, the appropriate extract liquids were added to the medium and replenished every 2 days. For Alizarin Red S (ARS) staining, the cells were fixed with 4% paraformaldehyde for 30 min and stained with 1% ARS (Leagene Biotechnology, China) after 14 days of induction.

#### Evaluation of angiogenic effects

HUVECs (2 × 10⁴ cells/well) were suspended in DMEM containing 1% FBS supplemented with the extract liquids of HAMA-CPC, HAMA-CPC@2D-sEVs, or HAMA-CPC@3D-sEVs. Matrigel (Corning, USA) mixed with 50% DMEM was pipetted into a 96-well plate on ice and allowed to gel in an incubator at 37 °C for 30 min. HUVECs were then seeded on the Matrigel surface and incubated for 2 h at 37 °C. Images of the formed endothelial tubes were captured via a microscope and analyzed with ImageJ software.

The fertilized chicken eggs were incubated at 38 °C with 80% humidity. On day 3 of embryo development, a small window was created in the eggshell under aseptic conditions and resealed. On day 9, chorioallantoic membranes (CAMs) were treated with 200 µL of the extract liquids of HAMA-CPC, HAMA-CPC@2D-sEVs, or HAMA-CPC@3D-sEVs. After 5 days of incubation, the blood vessels in the treated area were photographed with a stereomicroscope, and the number and length of the blood vessels were quantified via ImageJ software.

#### Quantitative real-time PCR analysis (qRT-PCR)

Total RNA was isolated via TRIzol reagent (Invitrogen, USA) and the Tiangen MiRcute Kit (Tiangen, China). The RNA concentration and quality were measured with a NanoDrop 2000 spectrophotometer (Thermo Fisher, USA). cDNA was synthesized from 20 µL of RNA via a high-capacity cDNA synthesis kit (Applied Biosystems, USA). qRT‒PCR was performed with SYBR Green PCR Master Mix (Thermo Fisher, USA) on a CFX Connect Real-Time RT‒PCR System (Bio-Rad, USA). The expression of collagen type I (COL-1), osteocalcin (OCN), Runx2, vascular endothelial growth factor (VEGF), CD31, and S1PR1 was quantified, with GAPDH as the endogenous control. The relative changes in gene expression were determined via the 2 − ΔΔCt method, and all analyses were conducted in triplicate.

#### Western blot analysis

Proteins from cells, sEVs, and tissues were lysed in ice-cold RIPA buffer (Beyotime, China) containing a protease inhibitor cocktail (Sigma, USA). The protein concentration was quantified via a BCA protein assay kit. Equal amounts of protein (40 µg per sample) were loaded and separated via SDS‒PAGE for 90 min and then transferred to PVDF membranes (Millipore, USA) on ice for 90 min. The membranes were blocked with 5% BSA for 1 h at room temperature. After blocking, the membranes were incubated overnight at 4 °C with primary antibodies against COL-1 (Proteintech, USA) and OCN (Santa Cruz Biotechnology, USA) for BMSCs; VEGF (Proteintech, USA), CD31 (Abcam, USA), and S1PR1 (Abcam, USA) for HUVECs; and CD63, CD81, TSG101 (Abcam, USA), calnexin (Proteintech, USA) and NAMPT (Abcam, USA) for sEVs. After three washes with TBST, the membranes were incubated with HRP-conjugated secondary antibodies (Abcam, USA) for 1 h at room temperature. The protein bands were visualized via an enhanced chemiluminescence substrate (NCM Biotech, China) and captured via a Chemidoc™ MP Imaging system (Bio-Rad, USA). The intensity of the protein bands was quantified via Image-Pro Plus 6.0 software and normalized to the levels of β-actin or GAPDH before comparison.

#### Immunofluorescence staining

The cells were fixed in 4% paraformaldehyde for 30 min at room temperature, followed by three washes with PBS. The cells were then blocked with normal goat serum at room temperature for 30 min. After blocking, the cells were incubated with primary antibodies specific for COL-1 (Proteintech, USA) for BMSCs and VEGF (Proteintech, USA) for HUVECs at 4 °C for 8–16 h. Following three washes with PBS, the cells were incubated with species-matched Alexa Fluor-conjugated secondary antibodies (Invitrogen, USA) in the dark for 1 h. The nuclei were counterstained with DAPI (Thermo Fisher, USA) in the dark for 5 min. Fluorescence images were acquired via a confocal microscope (Carl Zeiss, Germany).

### Animal experiments

SD rats (male, 10 weeks old, approximately 250 g) were purchased from the Guangdong Medical Laboratory Animal Center. All animal care and procedures were conducted in accordance with the Animal Protection and Use Regulations of the Third Affiliated Hospital of Sun Yat-sen University (approval no: SYT2024034).

#### Rat tibial drilling model

Under anesthesia with 2.5% isoflurane, the bilateral lower limbs of the rats were shaved, and the skin was disinfected with type III povidone-iodine. A 2 cm medial longitudinal incision was made at the proximal end of each tibia, and the skin, fascia, and muscle tissues were dissected to expose the medial aspect of the proximal tibia. Defects were drilled into the medial tibial plateau approximately 0.5–1 cm below the joint line via a 2.5 mm drill bit to a depth of 3 mm. After washing with PBS, the defects were filled with approximately 200 µL of hydrogel, ensuring complete defect coverage. The excess hydrogel was removed, and the hydrogel was cured via UV light exposure for 10 s. The incisions were sutured sequentially, and postoperative warmth was maintained via an electric heating blanket until the rats recovered from anesthesia. Postoperative care included oral antibiotics and analgesics for three days. The rats were euthanized at 2- and 4- weeks post-surgery under 10% chloral hydrate anesthesia. Following euthanasia, the tibial samples were harvested, and various analyses were conducted.

#### Micro-CT scanning

For micro-CT scanning, the specimens were aligned along their longitudinal axes in a custom holder within the micro-CT scanner (ZKKS-MCT-Sharp). The scanning protocol was set with a voltage of 70 kV and a power of 7 W, incorporating a 4-frame superposition to enhance image quality. The scans were performed with an angle increment of 0.72 degrees and an exposure time of 100 ms, completing a full 360-degree rotation to capture detailed images. The quantitative analysis focused on the bone volume (BV), bone volume-to-tissue volume ratio (BV/TV), and trabecular thickness (Tb.Th), providing detailed insights into the microarchitecture within the defect areas.

#### Histological analysis

The fixed samples were decalcified in 10% EDTA solution with gentle agitation for one month. After decalcification, the samples were dehydrated through an increasing series of ethanol concentrations (70%, 80%, 90%, 95%, and 100%) for optimal tissue preservation. After dehydration, the samples were embedded in paraffin and sectioned into 5 μm slices. For histological evaluation, the sections were subjected to hematoxylin and eosin (H&E) staining to assess the general tissue structure and Masson’s trichrome stain (MST) to evaluate collagen fiber deposition and bone matrix organization.

#### Immunohistochemical analysis

Tissue Sect. (5 μm) were deparaffinized in environmentally friendly solutions followed by rehydration through a graded series of ethanol. After the samples were rinsed with distilled water, antigen retrieval was performed via pepsin at 37 °C for 30 min. The sections were then washed with PBS and treated with 3% hydrogen peroxide to block endogenous peroxidase activity for 25 min. Blocking was conducted with 3% bovine serum albumin (BSA) at room temperature for 30 min. Primary antibodies targeting OCN and COL-1 for osteogenic assessment and VEGF and CD31 for angiogenic evaluation were applied, and the samples were incubated overnight at 4 °C. After PBS washes, horseradish peroxidase (HRP)-conjugated secondary antibodies were applied at room temperature for 50 min. Visualization was achieved via the use of diaminobenzidine (DAB) with a controlled staining time, followed by hematoxylin counterstaining. The sections were then dehydrated in an ascending alcohol series and cleared in xylene before being mounted with coverslips.

### Proteomic analysis

sEVs or cells from each group were collected and stored at -80 °C before being sent for quantitative proteomic analysis. Two separate analyses were conducted: the first analyzed extracted 2D-sEVs and 3D-sEVs, while the second focused on HUVECs treated with these sEVs. For both analyses, proteins were extracted via lysis buffer, followed by sonication and centrifugation to collect the supernatant. The extracted proteins underwent reduction, alkylation, and trypsin digestion, and the resulting peptides were desalted for liquid chromatography with tandem mass spectrometry (LC‒MS/MS) analysis. Peptide separation was performed on a reversed-phase column via an Easynano 1200 system, with mass spectrometry conducted on a Faims-Fusion instrument in DDA mode. Protein identification and quantification were achieved using ProteinDiscovery software by searching against the *Macaca mulatta* database. Bioinformatic analyses included Gene Ontology (GO), Kyoto Encyclopedia of Genes and Genomes (KEGG) pathway, and domain annotation via relevant databases, with functional enrichment assessed via Fisher’s exact test (*P* < 0.05). Protein‒protein interaction (PPI) networks were constructed via STRING, and visualizations were created with R packages for clustering and heatmap analysis.

### Statistical analysis

The data in this study are presented as the means ± standard deviations (SDs). All the statistical analyses were performed via GraphPad Prism software. For comparisons between two groups, Student’s t test was utilized. For comparisons involving more than two groups, one-way ANOVA was employed. Significance levels were set as follows: * for *P* < 0.05 and ** for *P* < 0.01. Differences were considered statistically significant at P values less than 0.05.

## Results

### Identification and characterization of 3D-sEVs

**Fig. 1:**
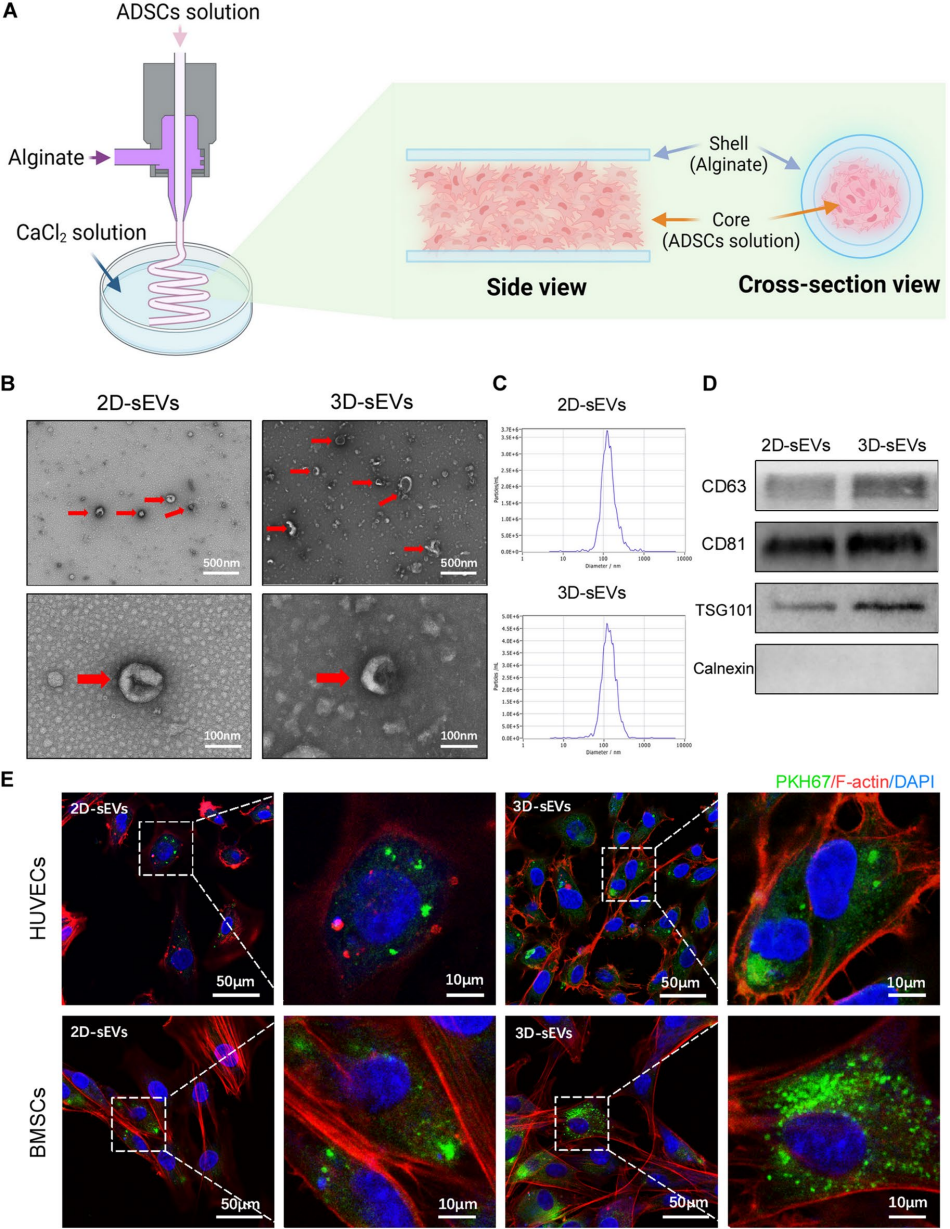
Identification of sEVs of ADSCs cultured by 3D coaxial bioprinting. (**A**) Schematic diagram of cellular microfiber structure fabricated based on coaxial 3D bioprinting. (**B**) The morphology of 2D-sEVs and 3D-sEVs evaluated by TEM. (**C**) NTA analysis showing the size distribution of 2D-sEVs and 3D-sEVs. (**D**) Western blot analysis showing expression levels of sEVs markers CD63, CD81 and TSG101 in 2D-sEVs and 3D-sEVs. (**E**) Laser confocal microscopy images showing the internalization of fluorescently labeled 2D-sEVs and 3D-sEVs by HUVECs and BMSCs

In this study, ADSCs and alginate solution were coaxially extruded to produce dense 3D microfibers for cultivation, and the 3D-sEVs were subsequently extracted by enriching the culture supernatant (Figs. 1A, S1A-B). Quantitative analysis of sEVs production (Figure S1C), consistent with our previous research findings [26], confirmed the high-yield advantage of the 3D microfiber culture system. TEM revealed the distinctive cup-shaped morphology of the 2D-sEVs and 3D-sEVs (Fig. 1B). Furthermore, NTA demonstrated that 2D-sEVs and 3D-sEVs had a uniform size distribution (Fig. 1C). Western blotting analysis confirmed the expression of the sEVs-specific markers CD63, CD81, and TSG101 in 2D-sEVs and 3D-sEVs, while the absence of calnexin (an endoplasmic reticulum secretory protein) was also noted (Fig. 1D). For observation of the internalization of sEVs by HUVECs and BMSCs, which is a prerequisite for their functional activity, the cells were incubated with PKH67-labeled sEVs for 24 h. Confocal microscopy revealed that 2D-sEVs and 3D-sEVs were predominantly internalized and localized in the perinuclear region of HUVECs and BMSCs, which demonstrated effective cytoplasmic uptake and high internalization efficacy (Fig. 1E).

### Characterization of HAMA-CPC hydrogels

**Fig. 2:**
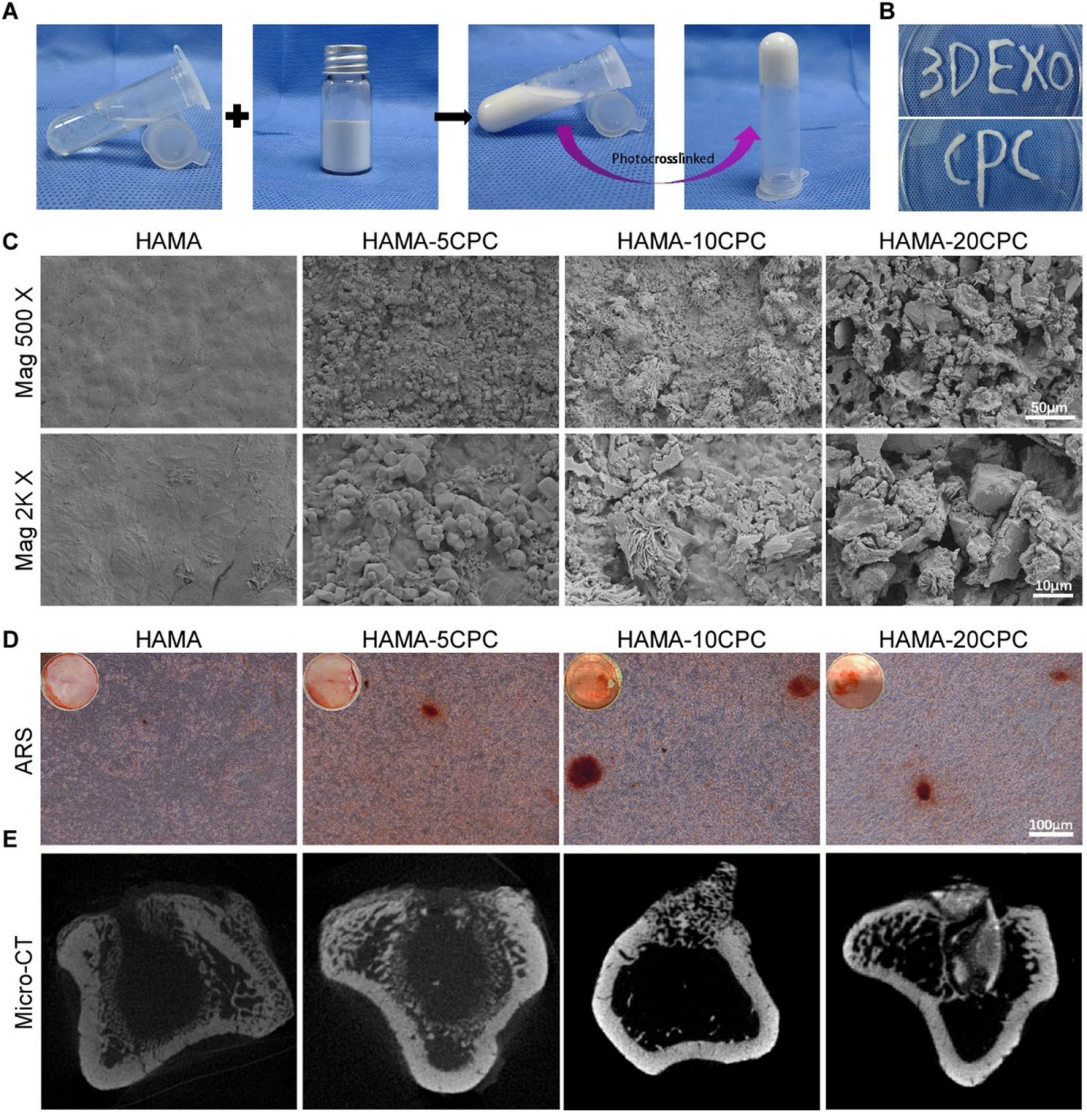
Physicochemical properties of HAMA-CPC. (**A**) HAMA, CPC, a flowable hydrogel composite HAMA-CPC, and a solid hydrogel obtained through photocrosslinking. (**B**) Photograph of injectable HAMA-CPC writing letters through a syringe. (**C**) SEM images of HAMA, HAMA-5CPC, HAMA-10CPC and HAMA-20CPC. (**D**) Alizarin red staining in co-culture for 14 days. (**E**) MicroCT images of the tibia 4w after material implantation

HAMA was synthesized through the introduction of a methacryl group into the molecular chain of hyaluronic acid, thereby endowing it with light-curing ability. The chemical molecular structure is presented in Figure S2A. In its solid state, CPC appears as a finely powdered white substance, and after combination with HAMA, the resulting mixture forms a milky white colloidal solution with fluidity before photo-crosslinking (Fig. 2A). The incorporation of CPC into 10% HAMA solution didn’t significantly affect the photo-curing property of the hydrogel. It could transform into a hydrogel under UV light exposure within about 15 s, similar to the unmodified HAMA solution (Figure S2B). FTIR spectral analysis verified the successful synthesis of the composite hydrogel, with distinct new absorption peaks emerging at approximately 566 cm⁻¹ and 1068 cm⁻¹, suggesting the presence of CPC. Importantly, these peaks became more prominent as the concentration of CPC increased (Figure S2C).

Rheological studies demonstrate that the composite hydrogels possess excellent viscoelasticity and injectability, with the storage modulus (G’) consistently surpassing the loss modulus (G”) across the tested frequency range. This stability indicates that the hydrogels primarily exhibit elastic behavior and structural integrity. Notably, HAMA − 10CPC displayed higher G’ and G” values than the other samples, suggesting that the addition of 10% CPC enhances viscoelastic properties (Figure S2D). Both G’ and G” increased compared to HAMA, particularly in the low shear strain region (Figure S2E), where high viscosity indicates strong cohesion and stability in static or slow-flowing conditions (Figure S2F). Additionally, the viscosity of all samples decreased with increasing temperature, with HAMA − 10CPC showing a relatively high initial viscosity and moderate changes in response to temperature variations (Figure S2G). This enhanced viscoelasticity and stability contribute to the hydrogel’s excellent injectability, allowing for precise delivery through a 1 mL syringe needle, as demonstrated by the ability to write letters with high accuracy (Fig. 2B).

SEM observations indicated that the surface of HAMA was smooth, whereas the surface of the composite hydrogel prepared with the addition of CPC became rough. In the HAMA-5CPC sample, sparsely distributed CPC particles were observed on its surface. Notably, the HAMA-10CPC sample presented needle-like particles and irregular small crystals. The HAMA-20CPC sample exhibited a morphology characterized by larger and denser CPC particles (Fig. 2C).

To evaluate the osteogenic effects of CPC in the hydrogel matrix, we performed ARS staining. Compared with the control group with the HAMA hydrogel, the HAMA-5CPC group presented more ARS-positive staining, with an even greater increase observed in the HAMA-10CPC group. However, further increasing the CPC concentration to 20% did not result in a significant increase in the ARS-positive areas (Figures. 2D, S3A). The osteogenic potential of the composite hydrogel was further assessed in vivo via a Sprague–Dawley rat tibial burr hole model. Micro-CT scans conducted four weeks post-operation indicated that the HAMA-10CPC group had the highest level of new bone formation (Fig. 2E, S3B). In contrast, the HAMA-20CPC group presented reduced bone formation, likely due to excessive residual CPC (Figure S3C-D). On the basis of these findings, the HAMA-10CPC formulation was chosen for further studies as sEVs carrier.

**Fig. 3:**
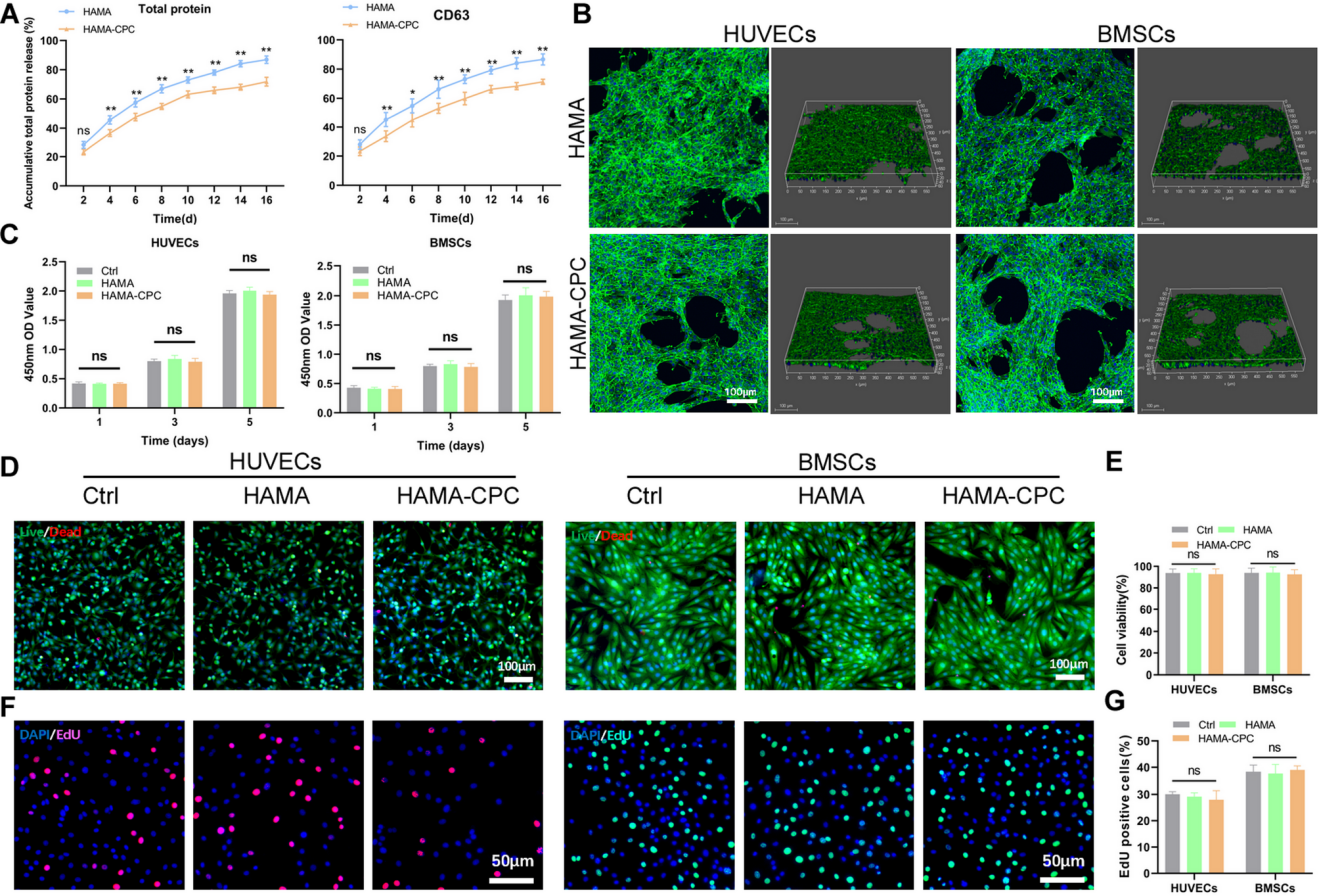
Characterization of HAMA-CPC@3D-sEVs. (**A**) Release profiles of total protein and CD63 of sEVs in HAMA and HAMA-CPC. (**B**) Laser confocal observation of the growth morphology of HUVECs and BMSCs in HAMA and HAMA-CPC. (**C**) CCK-8 assay of HUVECs and BMSCs treated with HAMA and HAMA-CPC. (**D**) Live/Dead assay images and (**E**) quantitative analysis. (**F**) EdU assay images and (**G**) quantitative analysis

Release analysis using both the BCA method and CD63 ELISA method indicated that sEVs could still be detected 16 days after application in both the HAMA and HAMA-CPC groups. The results from the CD63 ELISA, consistent with the BCA detection, showed that the HAMA-CPC hydrogel resulted in a relatively lower rate of sEVs release than did the pure HAMA hydrogel in vitro (Fig. 3A). According to the confocal images (Fig. 3B), HUVECs and BMSCs cultured on both HAMA and HAMA-CPC hydrogels exhibited a well-spread morphology, being flat and elongated with visible cell-cell contacts. The relatively uniform cell density indicated that the cells could attach and grow favorably on both substrates. Subsequently, CCK-8 assays, live-dead staining, and EdU assays were conducted. Neither the HAMA nor the HAMA-CPC hydrogels exhibited significant cytotoxic effects (Fig. 3C - G).

### Effects of HAMA-CPC@3D-sEVs on the proliferation, migration, and osteogenic differentiation of BMSCs

First, to assess the osteogenic potential of the HAMA-CPC composite hydrogel containing 3D-sEVs (HAMA-CPC@3D-sEVs), we used the extract to treat BMSCs. The proliferative response of the BMSCs to the 3D-sEVs was assessed via a CCK-8 assay. The results demonstrated that 3D-sEVs significantly increased cell proliferation in a dose-dependent manner on days 3 and 5 (Figure S4A). On days 3 and 5, the BMSCs treated with HAMA-CPC@3D-sEVs presented higher OD values than those treated with HAMA-CPC@2D-sEVs did (Figure S4B).

**Fig. 4:**
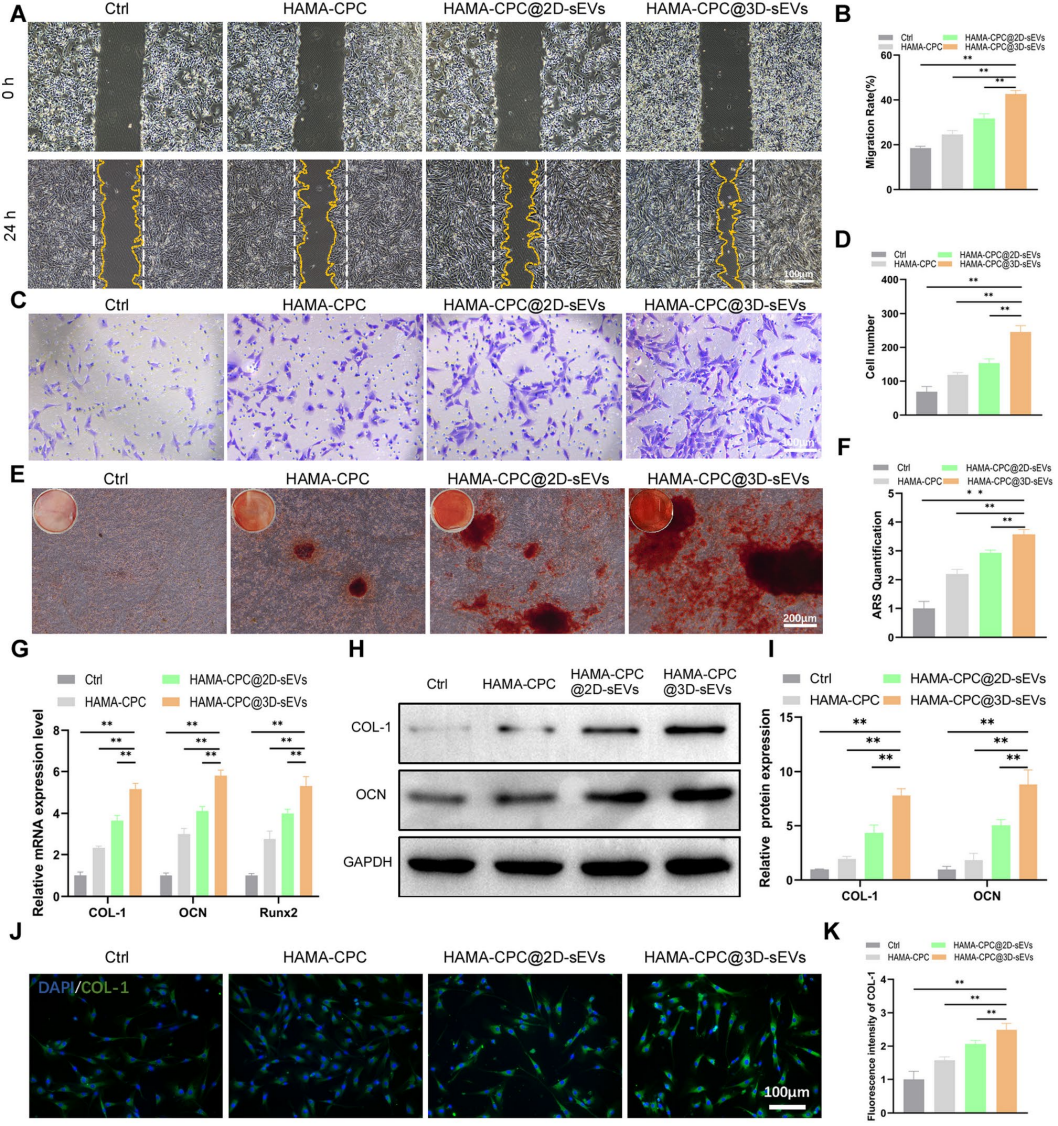
Effects of HAMA-CPC@3D-sEVs on BMSC Migration, and Osteogenic Differentiation. (**A**) Wound healing assay of BMSCs and (**B**) quantitative analysis. (**C**) Transwell assay images and (**D**) quantitative analysis of BMSC migration at 24 h. (**E**) Alizarin Red staining images and (**F**) quantitative analysis of mineralized matrix formation. (**G**) qRT-PCR analysis of osteogenesis-related mRNA expression (COL-1, OCN, and Runx2) in BMSCs. (**H**) Western blot images and (**I**) quantitative analysis of osteogenic protein expression (COL-1 and OCN) in BMSCs. (**J**) Immunofluorescence staining images of COL-1 expression and (**K**) corresponding quantitative analysis in BMSCs treated with HAMA-CPC@3D-sEVs

Second, to assess the in-situ bone regenerative potential, which depends on the migration of endogenous BMSCs to promote tissue remodeling, we conducted wound healing and Transwell assays. The results of the wound healing and Transwell assays revealed that HAMA-CPC@3D-sEVs significantly increased BMSC migration in both the horizontal and vertical directions (Fig. 4A-D). After 14 days of incubation in OM with either HAMA-CPC@2D-sEVs or HAMA-CPC@3D-sEVs, ARS staining and quantitative analysis demonstrated increased mineralization, with a more prominent effect in the 3D-sEVs group (Fig. 4E, F).

Finally, qRT‒PCR analysis further revealed that HAMA-CPC@3D-sEVs induced the upregulation of osteogenic genes, including COL-1, OCN, and Runx2 (Fig. 4G). Western blot analysis confirmed the increased expression of osteogenic proteins (COL-1 and OCN) in the HAMA-CPC@3D-sEVs group (Fig. 4H, I). Immunofluorescence staining revealed the strongest COL-1 fluorescence intensity in the HAMA-CPC@3D-sEVs group (Fig. 4J, K), highlighting the superior osteogenic potential of 3D-sEVs within this composite hydrogel system.

### Effects of HAMA-CPC@3D-sEVs on the proliferation, migration and angiogenesis of HUVECs

Wound healing and Transwell assays confirmed that HAMA-CPC@3D-sEVs strikingly increased the migration velocity and of the number of migrating HUVECs when they were combined with HAMA-CPC@2D-sEVs (Figure S5A-D). The HAMA-CPC@3D-sEVs cluster demonstrated a proliferative effect on HUVECs similar to that observed in BMSCs, with the peak OD measured at 450 nm on the 3rd and 5th days (Figure S5E).

**Fig. 5:**
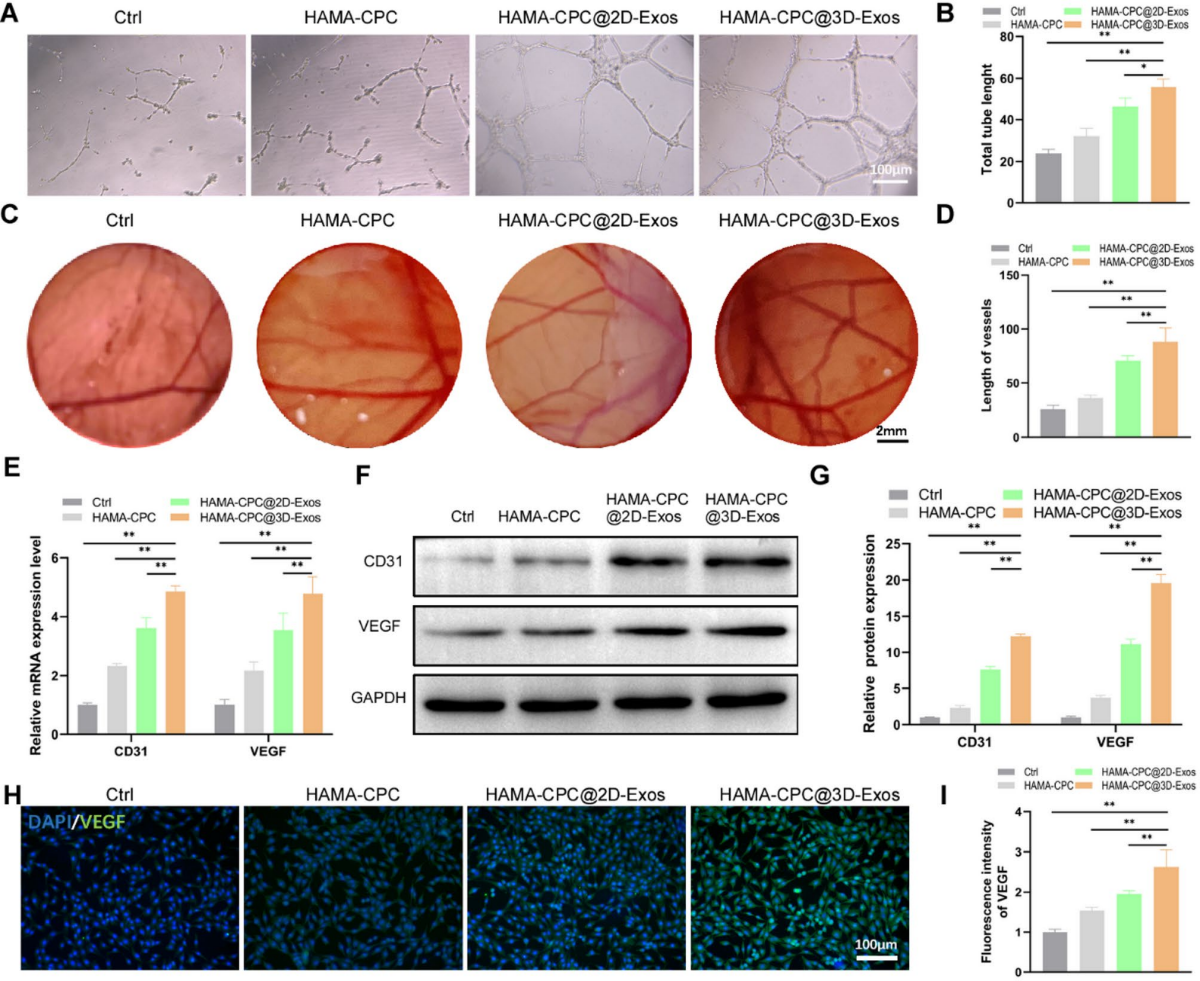
Effects of HAMA-CPC@3D-sEVs on HUVEC angiogenesis. (**A**) Tube formation assay images and (**B**) quantitative analysis of total tube length. (**C**) CAM assay images and (**D**) quantitative analysis of total vessel length. (**E**) qRT-PCR analysis of angiogenesis-related mRNA expression (CD31 and VEGF) in HUVECs. (**F**) Western blot images and (**G**) quantitative analysis of CD31 and VEGF protein expression in HUVECs. (**H**) Immunofluorescence staining images of VEGF expression and (**I**) corresponding quantitative analysis

The proangiogenic ability of HAMA-CPC@3D-sEVs was determined via a tube formation assay involving HUVECs. Compared with HAMA-CPC@2D-sEVs, HAMA-CPC@3D-sEVs substantially increased tube formation, increasing the total tube length (Fig. 5A, B). Additionally, the angiogenic efficacies were corroborated in vivo via a chick embryo CAM assay. Consistent with the in vitro results, the CAM results revealed increased vessel genesis and an elongated total vessel length within the sEVs-treated assemblages, with HAMA-CPC@3D-sEVs showing the strongest effects (Fig. 5C, D).

Furthermore, HAMA-CPC@3D-sEVs markedly increased the mRNA and protein expression of the angiogenic biomarkers VEGF and CD31, as shown by qRT‒PCR (Fig. 5E) and Western blot analyses (Fig. 5F, G). Similarly, immunofluorescence staining revealed the most intense increase in VEGF fluorescence in the HAMA-CPC@3D-sEVs group (Fig. 5H, I), further confirming the increased angiogenic potential of this composite hydrogel system.

### HAMA-CPC@3D-sEVs promote osteogenesis and angiogenesis in vivo

**Fig. 6:**
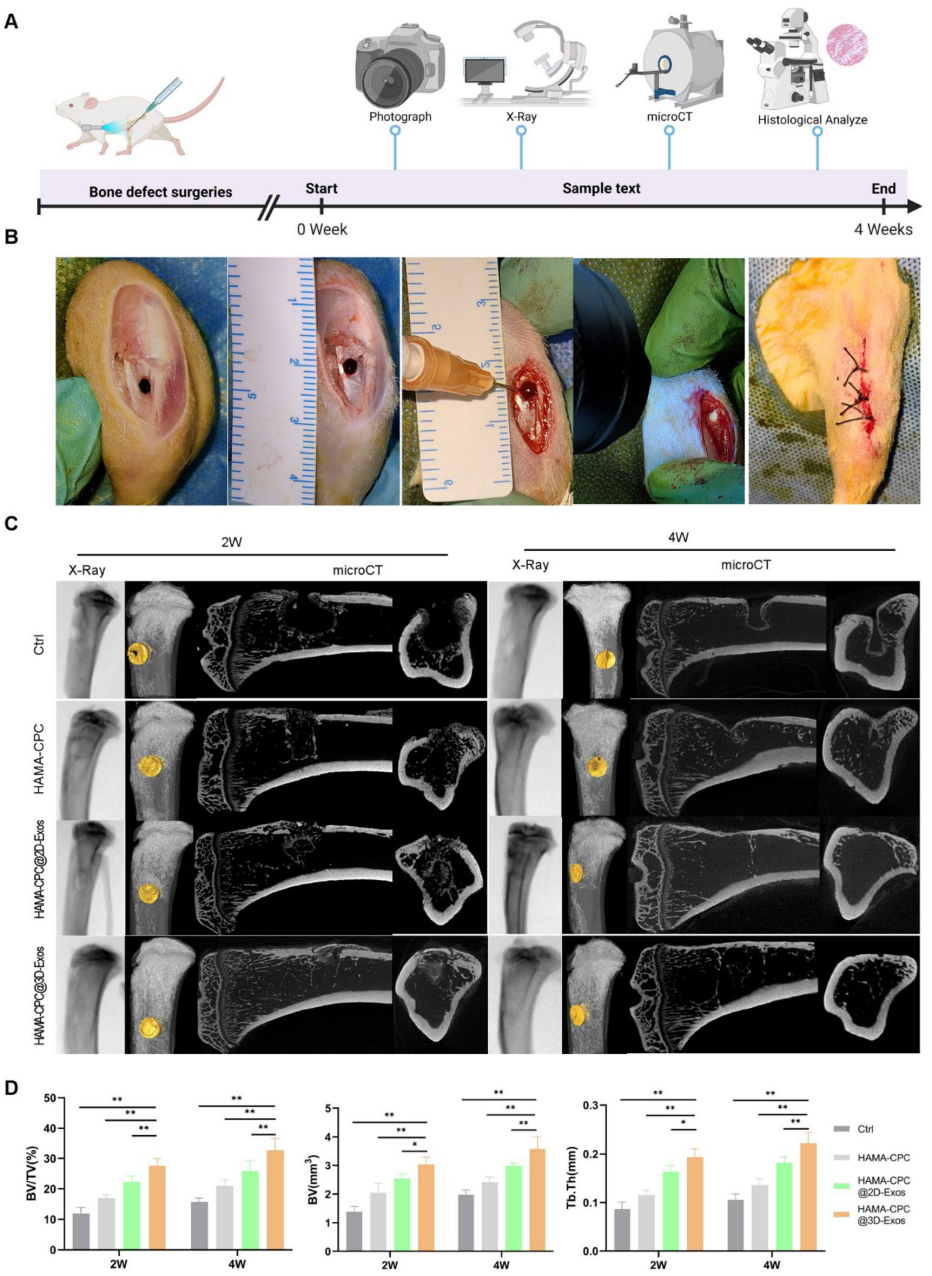
The effect of HAMA-CPC@3D-sEVs on bone regeneration in rat tibial defect model. (**A**) Schematic of animal experiment processes. (**B**) Photographs of surgical procedures of the rat tibial drilling model. (**C**) Representative images of 2- and 4-week radiographs and micro-CT. (**D**) Quantitative analysis of bone regeneration by using BV/TV(%), BV(mm^3^) and Tb.Th(mm)

To conduct an in-depth in vivo assessment of the osteogenic and angiogenic potential of the injectable HAMA-CPC@3D-sEVs, we employed a rat tibial defect model. The experimental design is delineated in a schematic diagram (Fig. 6A). In rats, a 2.5 mm tibial defect was surgically created, and subsequently, the HAMA-CPC@3D-sEVs hydrogel was precisely injected into the defect site (Fig. 6B). Radiographic and micro-CT analyses, which were carried out at 2- and 4- weeks post-injection, revealed progressive bone healing (Fig. 6C). Notably, compared with the control group, the HAMA-CPC@3D-sEVs group exhibited substantially increased bone regeneration at both time intervals. Quantitative micro-CT analysis further confirmed these observations, revealing significantly greater BV/TV, BV, and Tb. Th in the HAMA-CPC@3D-sEVs group than in the control groups at both 2- and 4- weeks post-surgery (Fig. 6D).

**Fig. 7:**
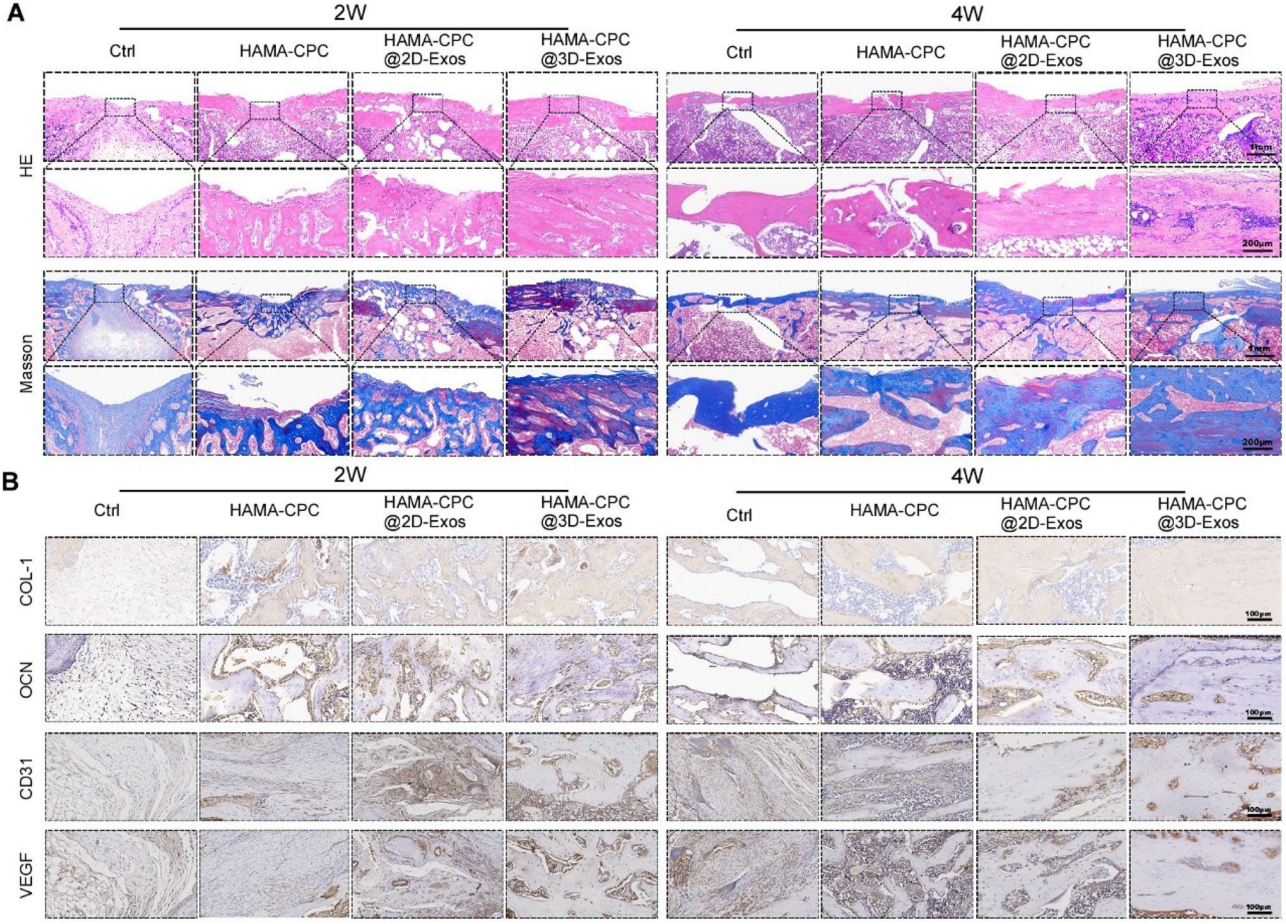
Histological evaluation of bone formation at 2 and 4 weeks. (**A**) Representative HE and Masson’s trichrome staining images. (**B**) Representative immunohistochemical staining images for COL-1, OCN, CD31, and VEGF

Histological examination of tibial defect tissue sections was performed. At 2 weeks post-surgery, HE and Masson’s trichrome staining revealed minimal fibrous tissue in the blank group. In contrast, the HAMA-CPC and HAMA-CPC@2D-sEVs groups demonstrated nascent neovascularization and new bone formation within the defect area and callus. The HAMA-CPC@3D-sEVs group, however, exhibited more extensive neovascularization, greater amounts of new bone tissue, and denser, more mature collagen fibers in the callus. By 4 weeks, the defects in the blank group were merely covered with a thin veneer of new bone tissue, whereas those in the HAMA-CPC and HAMA-CPC@2D-sEVs groups presented a thicker bone stratum. Notably, the HAMA-CPC@3D-sEVs group harbored the most substantial new bone tissue and the richest mature collagen extracellular matrix (ECM) (Fig. 7A).

Immunohistochemical staining for COL-1, OCN, CD31, and VEGF was performed (Fig. 7B). COL-1 staining intensified over time, with peak expression observed in the HAMA-CPC@3D-sEVs group at both 2 and 4 weeks. OCN expression was pronounced in mature bone tissue across all groups at both time points, with the HAMA-CPC@3D-sEVs group displaying the highest levels at 4 weeks. CD31 and VEGF, which serve as markers of vasculogenesis, were highly expressed at 2 weeks, with the greatest number of positive cells in the HAMA-CPC@3D-sEVs group. By 4 weeks, as the bone matured, the number of CD31- and VEGF-positive cells diminished.

### NAMPT contained in 3D-sEVs increases angiogenic effects

**Fig. 8:**
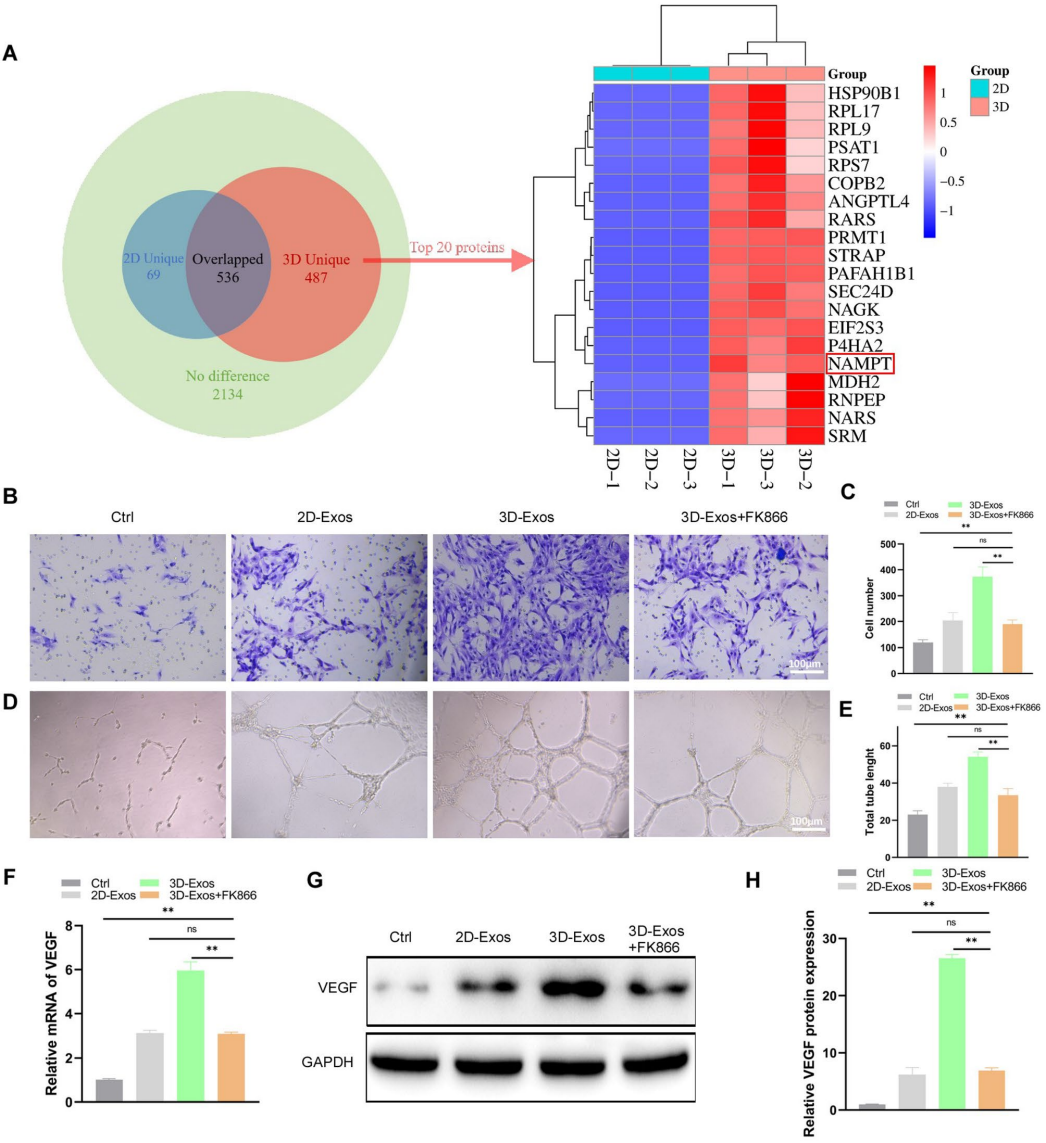
3D-sEVs enhance angiogenesis through NAMPT protein. (**A**) Venn diagram of detected in 3D-sEVs and 2D-sEVs (numbers represent the quantities of detected protein types) and heatmap of the top 20 uniquely expressed proteins in 3D-sEVs. (**B**) Transwell assay of HUVECs and (**C**) quantitative analysis. (**D**) Tube formation assay of HUVECs and (**E**) quantitative analysis. (**F**) qRT-PCR analysis of the expression levels of mRNA (VEGF) in HUVECs. (**G**) Western blot images and (**H**) quantitative analysis of VEGF expression in HUVECs

KEGG pathway analysis of 3D-sEVs-enriched proteins revealed significant associations with angiogenic and osteogenic processes (Figure S6A). Pro-angiogenic pathways included Rap1 signaling, Endocytosis, and Tight junction, while osteogenic regulation was linked to Parathyroid hormone signaling and MAPK pathway. GO analysis (Figure S6B) demonstrated that 3D-sEVs preferentially enriched proteins linked to angiogenesis, with biological processes (BP) highlighting endothelial cell migration, GTPase activity-mediated vascular morphogenesis, and extracellular matrix remodeling. Critical molecular functions (MF) included ATP-binding, protein-protein interactions, and metal ion binding, while cellular components (CC) featured extracellular exosomes, plasma membrane receptors, and cytoplasmic signaling hubs. Concurrently, osteogenesis-associated processes such as skeletal development and calcium ion-dependent mineralization were enriched, driven by DNA/RNA polymerase II-binding transcription factors and extracellular matrix deposition. This dual enrichment underscores the capacity of 3D-sEVs to coordinately regulate angiogenesis-osteogenesis coupling through transcriptional activation, metabolic reprogramming, and microenvironmental crosstalk, suggesting their therapeutic potential in vascularized bone regeneration. Given the critical role of angiogenesis in bone repair and regeneration, we further analyzed the upregulated proteins in 3D-sEVs to identify key mediators underlying their pro-angiogenic potential, which may synergistically enhance vascularized osteogenesis. Among the 3D-sEVs-specific proteins (*n* = 487), screening of the top 20 highly expressed candidates revealed NAMPT (Fig. 8A), a multifunctional enzyme with literature-supported roles in angiogenesis via endothelial cell activation and NAD + biosynthesis, suggesting its potential as a key mediator of 3D-sEVs-enhanced vascularization during bone repair. On the basis of these findings, we postulate that NAMPT serves as a pivotal mediator of the angiogenic effects induced by 3D-sEVs.

Western blot analysis confirmed the significant upregulation of NAMPT in 3D-sEVs (Figure S6C, D). When NAMPT was inhibited by FK866, the increase in HUVEC proliferation induced by 3D-sEVs was reversed (Figure S6E). Transwell migration assays demonstrated that FK866 also inhibited the promigratory effects of 3D-sEVs (Fig. 8B, C). Moreover, angiogenesis assays revealed that FK866 inhibited the proangiogenic activity of 3D-sEVs (Fig. 8D, E) as well as the concomitant increase in VEGF expression (Fig. 8F-H). These findings emphasize that the overexpression of NAMPT is a cardinal driving force behind the proangiogenic functions of 3D-sEVs.

### NAMPT in 3D-sEVs increases S1PR1 expression in HUVECs, leading to increased VEGF expression

To determine the molecular mechanism by which NAMPT in 3D-sEVs drives angiogenesis, we treated HUVECs with 2D-sEVs and 3D-sEVs via comparative proteomic analysis. The results revealed substantial alterations in protein expression after 3D-sEVs treatment compared to 2D-sEVs treatment, with 582 proteins exhibiting increased expression and 522 proteins showing decreased expression (Figure S7A). PPI analysis revealed a more intricate interaction web in the 3D-sEVs-treated cells, suggesting that 3D-sEVs promote a broader spectrum of cellular functions and signaling pathways (Figure S7B).

Density distribution analysis of protein abundance (Figure S8A) revealed a greater fraction of high-abundance proteins in the 3D-sEVs-treated cells than in the control cells, highlighting their increased capacity to increase protein expression. KEGG pathway analysis (Figure S8B) revealed angiogenesis-related pathways that were clearly influenced by 3D-sEVs, indicating increased endothelial cell proliferation, migration, and tube formation. GO enrichment analysis (Figure S8C) revealed that 3D-sEVs promoted biological processes related to angiogenesis, including cell proliferation, adhesion, signal transduction, and apoptosis inhibition. Additionally, cellular component analysis revealed enrichment of the ECM and membrane-associated components, suggesting a role in cell‒cell communication and signaling. Molecular function analysis revealed an increase in calcium ion and ATP-binding proteins, which are pivotal for endothelial migration, proliferation, and energy metabolism.

**Fig. 9:**
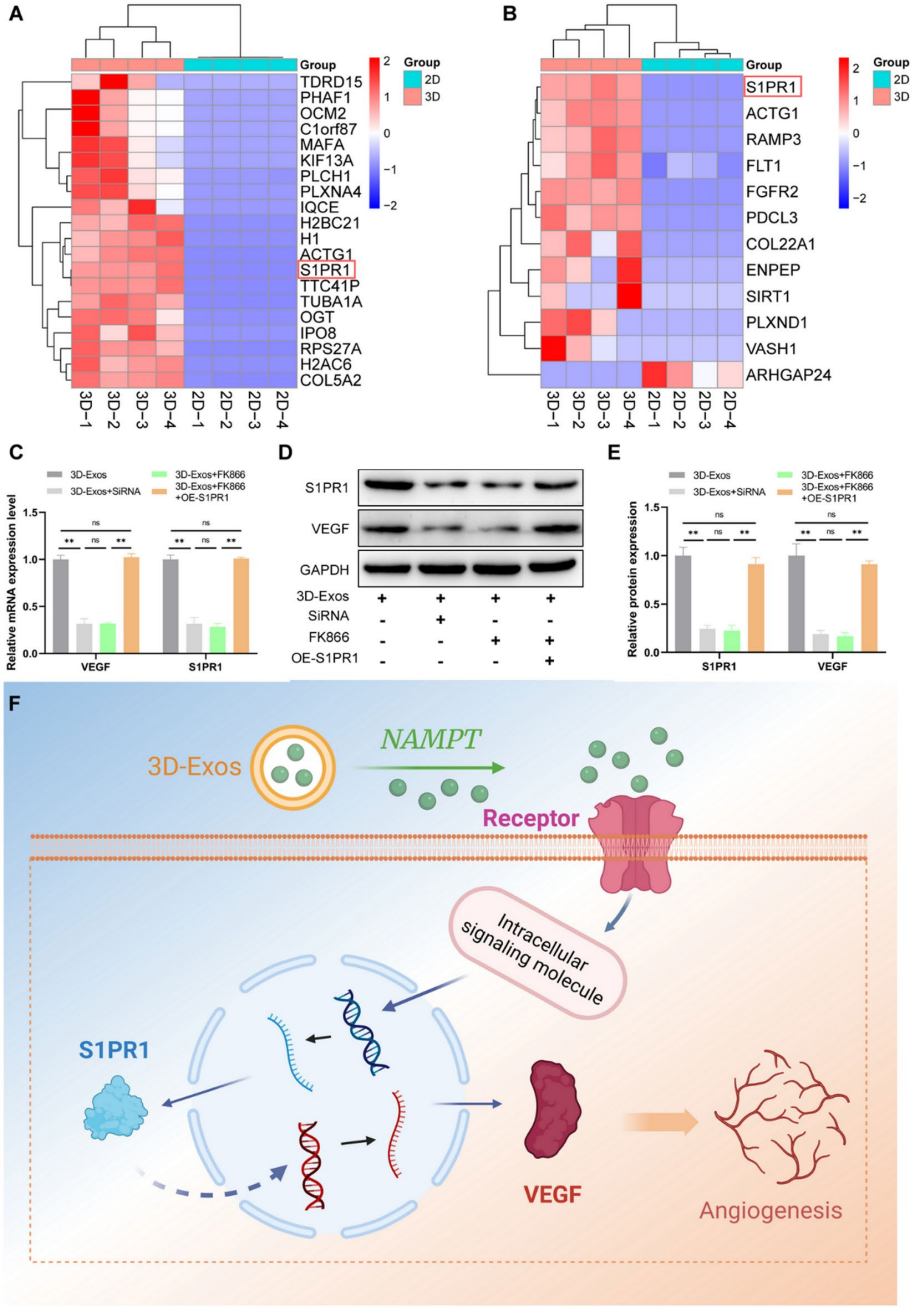
3D-sEVs function through the NAMPT-S1PR1-VEGF axis. (**A**) Heatmap of the top 20 differentially abundant proteins in HUVECs treated with 3D-sEVs versus 2D-sEVs. (**B**) Heatmap of differentially expressed proteins related to angiogenic biological processes. (**C**) qRT-PCR analysis of the expression levels of mRNA (S1PR1, VEGF) in HUVECs. (**D**) Western blot images and (**E**) quantitative analysis of S1PR1 and VEGF expression in HUVECs. (**F**) Schematic diagram of the 3D-sEVs functioning through the NAMPT-S1PR1-VEGF axis

Among the differentially expressed proteins, S1PR1 was among the top 20 most abundant in the 3D-sEVs-treated cells and was tightly linked to angiogenesis-related processes (Fig. 9A, B). Network analysis further highlighted S1PR1’s engagement in important processes such as cell proliferation, migration, and adhesion (Figure S9). To confirm the function of S1PR1, we performed siRNA-mediated knockdown of S1PR1 in HUVECs and found that this treatment strongly diminished both S1PR1 and VEGF expression at the mRNA and protein levels, mirroring the consequences of FK866 treatment. Conversely, S1PR1 overexpression reversed the FK866-induced suppression of S1PR1 and VEGF expression (Fig. 9C-E). These findings suggest that NAMPT in 3D-sEVs induces the upregulation of S1PR1 in HUVECs, thereby activating VEGF signaling and promoting angiogenesis (Fig. 9F).

## Discussion

Our study provides substantial insights into the repair of bone defects through the utilization of advanced bone tissue engineering strategies. Notably, we demonstrated that ADSCs-derived sEVs cultured in a 3D environment possess substantially greater osteogenic and angiogenic capabilities than those obtained from traditional 2D cultures. By integrating these 3D-sEVs into a composite hydrogel composed of HAMA and CPC, we fabricated a novel biomaterial with the capacity for sustained sEVs release and increased bone regeneration. This composite hydrogel not only increases the bioavailability of sEVs but also allows controlled release, thereby strongly facilitating the formation of both bone and vascular tissues in vivo. Collectively, these findings suggest that the HAMA-CPC hydrogel loaded with 3D-sEVs shows promise as a therapeutic approach for addressing bone defects.

Three-dimensional cell culture techniques can be broadly classified into scaffold-based and scaffold-free methodologies [20]. Scaffold-free methods rely on cellular adhesion mechanisms to form cell spheroids [21, 22], whereas scaffold-based techniques employ exogenous materials to mimic the natural ECM, providing a more organized environment for tissue engineering applications [37, 38]. Scaffold-based approaches, such as hydrogels, fibers, and porous scaffolds, promote cell–cell communication and proliferation [39, 40]. Compared with 2D cultures, 3D cultures are capable of yielding a greater quantity of sEVs by simulating a microenvironment that more closely resembles physiological conditions [22, 41]. However, current 3D culture methods typically achieve only a relatively modest increase in sEVs production, usually ranging from several to tens of times greater than that of 2D cultures [22, 38, 41]. Our coaxial bioprinting-based 3D culture platform overcomes this limitation by increasing the extracellular vesicle yield by approximately 1000-fold [26]. This substantial increase can be attributed to the encapsulation of high-density MSCs within micron-scale hydrogel fibers, which enables self-assembly, ECM secretion, and effective nutrient exchange. However, the significant increase in sEVs production by our 3D coaxial bioprinting platform raises concerns about the purity of derived EVs. TEM images (Fig. 1B) show a cleaner background in 2D-sEVs images and a more cluttered one in 3D-sEVs images, suggesting potential 3D-sEVs purity issues. The 3D culture’s high-density MSCs in hydrogel fibers create a complex microenvironment. While it boosts sEVs production, the enhanced cellular activity releases more cellular debris and by-products. These are more likely to co-isolate with 3D-sEVs during extraction, affecting the particle/protein ratio, an important purity indicator. Although our isolation protocol aims to minimize contaminants, the TEM results indicate room for improvement. Future studies will use advanced techniques for in-depth sEVs characterization, focusing on precisely quantifying the particle/protein ratio for 2D- and 3D-derived sEVs. We’ll also optimize isolation methods to reduce contaminants and enhance 3D-sEVs purity for reliable therapeutic applications.

MSC-derived sEVs have been the subject of extensive research, with a particular focus on those derived from bone marrow, adipose, and umbilical cord MSCs [38, 42]. Among these, ADSCs have attracted substantial attention because of their high availability, high survival rates, and multidirectional differentiation potential [43]. Compared with BMSC-derived sEVs, ADSC-derived sEVs have been shown to exhibit superior osteogenic and angiogenic properties [44]. For example, ADSC-sEVs promote the osteogenic differentiation of MSCs and promote fracture healing by transporting bioactive molecules such as RNAs, proteins, and lipids [45, 46]. Comparative studies have revealed that ADSC-sEVs possess higher levels of angiogenic factors and greater proangiogenic effects than BMSC-sEVs do, as evidenced by improved endothelial cell migration and proliferation [47]. Additionally, ADSC-sEVs display increased resistance to apoptosis and improved immunomodulatory properties, rendering them highly suitable for therapeutic applications [48]. In the present study, we selected ADSCs as the source for 3D-sEVs and utilized a coaxial bioprinting platform to produce a sufficient quantity of sEVs for bone repair. These 3D-sEVs exhibited robust osteogenic and angiogenic effects, further substantiating their potential for sEVs-based bone tissue engineering.

Three-dimensional-cultured sEVs have been applied in a diverse range of biomedical applications, including tumor therapy, tissue engineering, and regenerative medicine [39, 49]. For example, 3D-sEVs derived from human dermal fibroblasts have demonstrated increased antiaging effects both in vitro and in a nude mouse photoaging model, which has been attributed to elevated TIMP-1 levels and differentially expressed miRNAs [21]. Similarly, 3D-cultured umbilical cord MSC (UMSC)-sEVs have been shown to promote osteochondral regeneration by activating TGFβ1 and Smad2/3 signaling [50]. Additionally, 3D-cultured BMSC-sEVs significantly promoted osteogenic differentiation through YAP signaling and inhibited BMSC apoptosis [37]. These collective findings suggest that 3D-sEVs exhibit improved biological functions compared with their 2D counterparts. In our study, compared with 2D-sEVs, 3D-sEVs significantly increased the proliferation, migration, and osteogenic differentiation of BMSCs (Figures 4, S4). This finding was confirmed by increased calcium deposition, osteogenic marker expression, and ARS staining following coculture in OM. Moreover, 3D-sEVs exhibited stronger proangiogenic effects, as evidenced by increased tube formation in vitro and increased vessel number and length in CAM assays Figure 5. These results emphasize the superior osteogenic and angiogenic potential of 3D-sEVs, positioning them as promising candidates for bone repair applications.

Given that the process of bone repair is rather lengthy, it is necessary to have an appropriate carrier to achieve long-term sustained release and local action of sEVs. Hydrogels, such as the HAMA hydrogel selected in this study, offer ideal properties including biocompatibility, tunable physicochemical characteristics, and ECM-mimicking abilities, making them suitable for prolonged sEVs retention at bone defect sites [29, 51]. While HAMA alone provides a stable microenvironment for sEVs, its osteogenic efficacy is limited. To address this, we incorporated CPC into the HAMA hydrogel. CPC not only enhances osteogenesis through its inherent bioactivity and osteoconductivity [36] but also synergistically regulates sEVs release kinetics through physical encapsulation, dynamic ionic interactions, and degradation-mediated control. The porous structure of HAMA-CPC, characterized by CPC-derived needle-like particles and irregular crystals (Fig. 2C), creates a physical barrier that restricts sEVs diffusion, thereby extending their retention time at the defect site [52]. This spatial confinement is further reinforced by dynamic calcium-phosphate interactions: CPC releases Ca²⁺ during degradation, which forms electrostatic complexes with phospholipids on sEVs membranes [53], as evidenced by FTIR peaks at 566 cm⁻¹ and 1068 cm⁻¹ (Figure S2C). Simultaneously, the slower degradation rate of CPC compared to HAMA [30, 54] ensures gradual matrix remodeling, aligning with the in vivo observation that HAMA-CPC maintained structural integrity for 4 weeks post-implantation (Fig. 2E). This degradation-mediated release kinetics prevents abrupt sEVs loss while supporting new bone ingrowth, as reflected in the sustained sEVs release profile over 16 days (Fig. 3A). From a materials science perspective, the 10% CPC formulation achieves an optimal balance between osteogenic activity and material stability. While higher CPC concentrations (e.g., 20%) increase residual mass (Figure S3B-C) and impair bone regeneration, 10% CPC provides sufficient calcium/phosphate ions to stimulate osteogenesis [55] without obstructing vascularization or MSCs migration. Biologically, this composite system may synergize with sEVs to regulate osteogenic pathways (e.g., BMP/Smad, Wnt/β-catenin [56]), as supported by enhanced mineralization in vitro (Fig. 4E) and vivo (Fig. 7A).

Angiogenesis plays a crucial role in bone defect repair, as it provides essential factors for tissue regeneration and interacts with various cellular and molecular pathways to drive the healing process, as supported by multiple studies [57, 58]. Therefore, we sought to further explore the underlying mechanisms by which 3D-sEVs promote angiogenesis. Proteomic analysis revealed that NAMPT, a rate-limiting enzyme in NAD + biosynthesis [59], is significantly upregulated in 3D-sEVs and plays a central role in angiogenesis [60]. NAMPT catalyzes the conversion of nicotinamide to nicotinamide mononucleotide (NMN), a precursor of NAD+, which is critical for energy metabolism, cell survival, and repair processes [59, 61]. Elevated NAD + levels, which are mediated by NAMPT, increase endothelial cell proliferation, migration, and survival, processes that are essential for angiogenesis [62, 63]. In this study, the NAMPT inhibitor FK866 was used to demonstrate that NAMPT in 3D-sEVs directly contributes to the upregulation of VEGF expression and the promotion of angiogenic activity. The attenuation of VEGF expression following FK866 treatment highlights the pivotal role of NAMPT in the proangiogenic effects of 3D-sEVs. Further analysis identified S1PR1, a G protein-coupled receptor, as a key downstream effector in the NAMPT signaling pathway. S1PR1 is activated by sphingosine-1-phosphate (S1P) and plays a critical role in regulating endothelial cell proliferation, migration, and angiogenesis [64, 65]. This molecule achieves this goal by maintaining endothelial barrier integrity, promoting vascular patterning, and interacting with vascular endothelial (VE)-cadherin to facilitate vessel formation [66, 67]. In this study, S1PR1 inhibition in HUVECs reduced VEGF expression, whereas S1PR1 overexpression reversed the suppression of VEGF caused by FK866. These findings confirm that NAMPT upregulates S1PR1 expression, which subsequently activates VEGF signaling and promotes angiogenesis. This mechanistic understanding highlights the therapeutic potential of modulating NAMPT and S1PR1 activities in bone defect repair.

## Conclusions

In this study, we have conclusively demonstrated the remarkable potential of 3D-sEVs, which are derived from ADSCs cultured through coaxial 3D bioprinting, in facilitating bone defect repair. The HAMA-CPC@3D-sEVs composite hydrogel effectively increased BMSCs proliferation, migration, and osteogenic differentiation while promoting angiogenesis in HUVECs both in vitro and in vivo. In a rat tibial defect model, this composite biomaterial was found to significantly promote bone regeneration, confirming its therapeutic potential. Proteomic analysis revealed that NAMPT, which is highly abundant in 3D-sEVs, upregulates the expression of S1PR1. This subsequent activation of S1PR1 leads to the activation of VEGF signaling, ultimately driving angiogenesis. These findings elucidate the mechanism of the increased proangiogenic and osteogenic effects exhibited by 3D-sEVs and emphasize their potential as a highly promising strategy in the fields of bone tissue engineering and regenerative medicine.

## Electronic supplementary material

Below is the link to the electronic supplementary material.

Supplementary Material 1

## Data Availability

Data is provided within the manuscript or supplementary information files.
