# Supplementary material for: Injectable HAMA-CPC hydrogels loaded with high-yield 3D bioprinted adipose-derived stem cell small extracellular vesicles for increased bone repair
Source: J Nanobiotechnology. 2025 Jul 21;23:531. doi: 10.1186/s12951-025-03596-4 (PMC12278526; doi:10.1186/s12951-025-03596-4)
Supplement: Supplementary file 1 — Supplementary Material 1 [file 12951_2025_3596_MOESM1_ESM.docx]

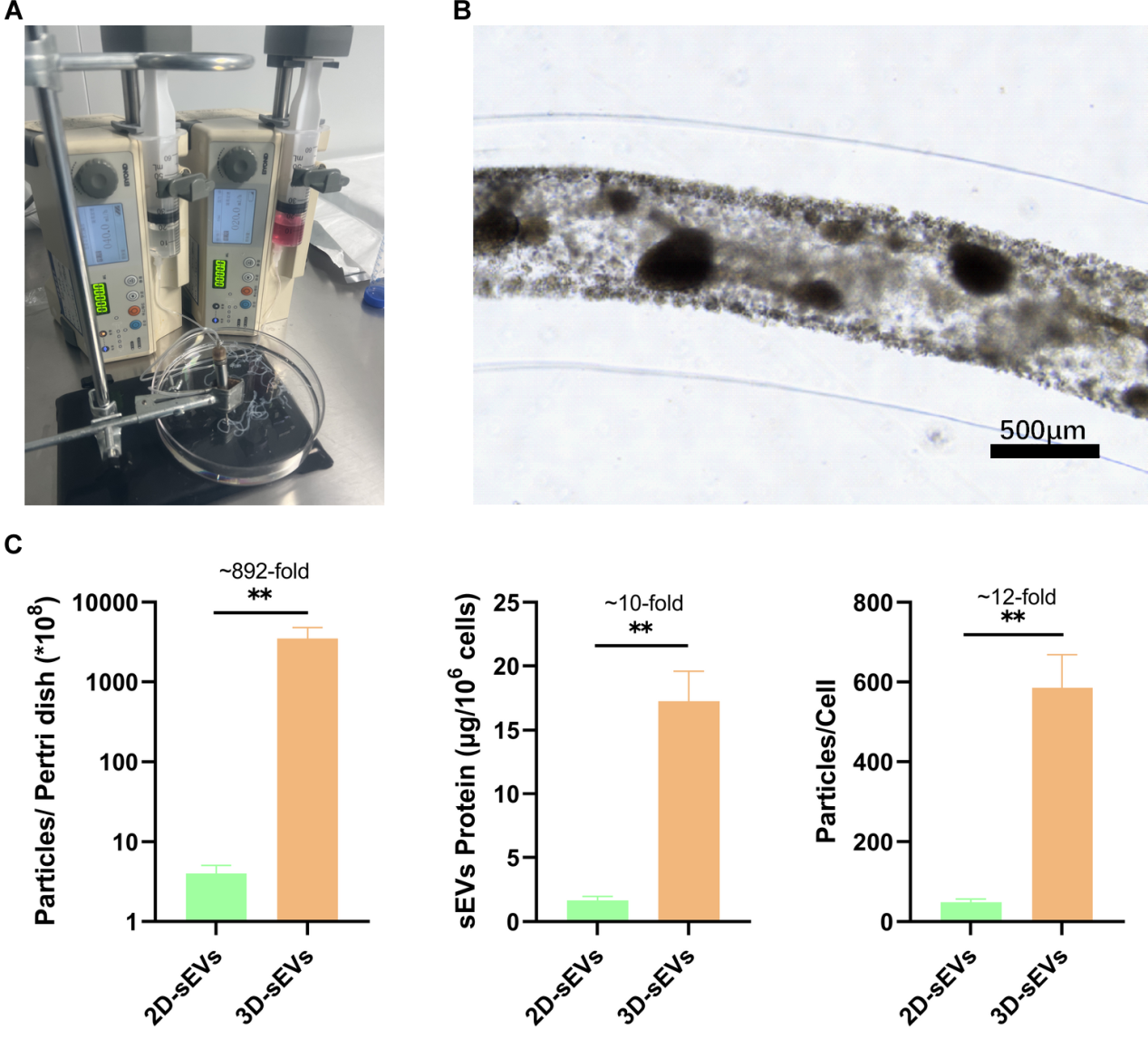


**Figure. S1.** (A) Image of cellular microfibers fabricated by coaxial 3D bioprinting. (B) Optical microscope image of printed cellular microfibers after 24 hours of cell culture. (C) Quantitative analysis of 2D and 3D sEVs production.


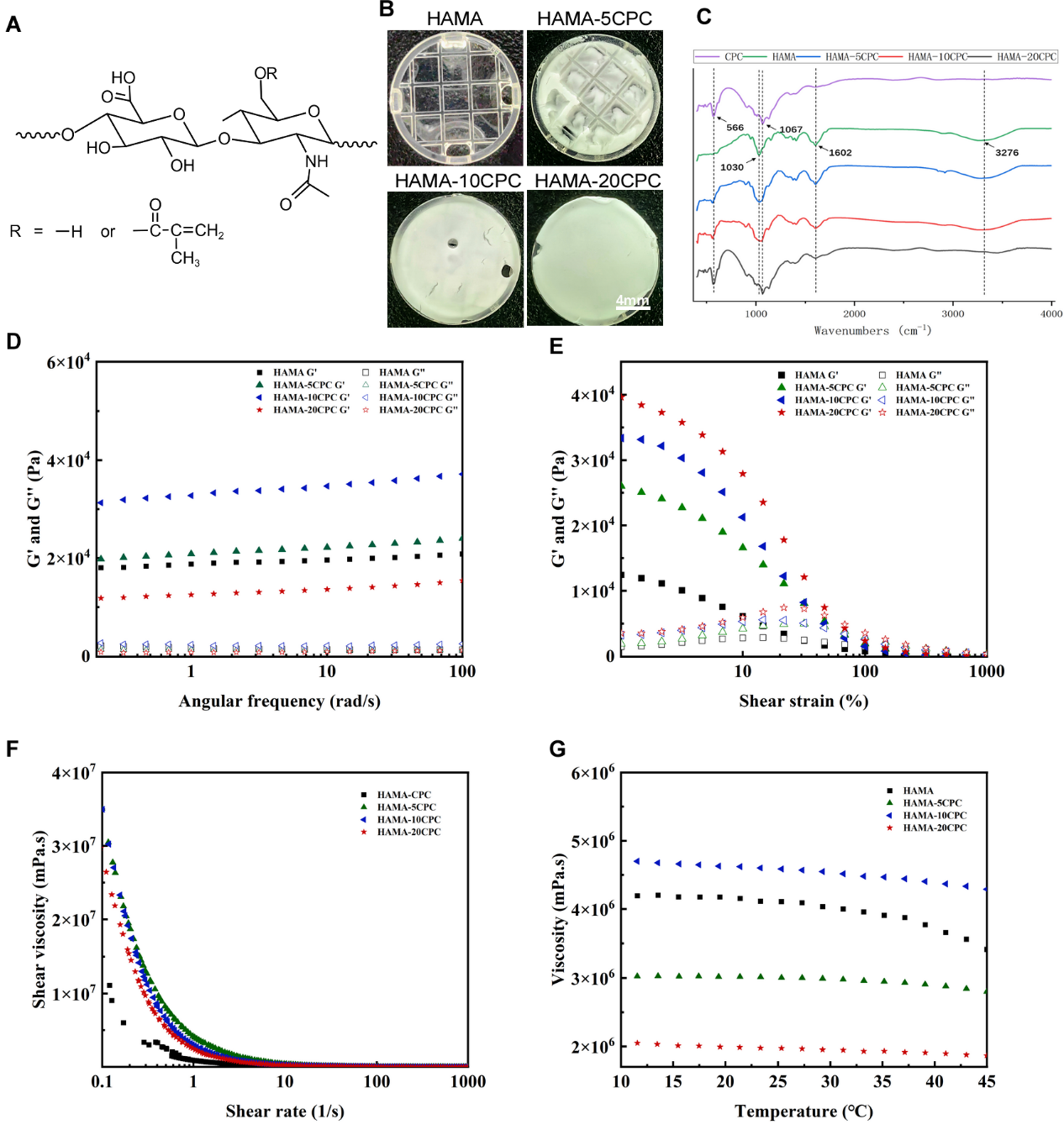


**Figure.S2.** (A) Chemical structure of HAMA. (B) Photographs of HAMA, HAMA-5CPC, HAMA-10CPC, and HAMA-20CPC on curing ring. (C) FTIR spectra of CPC, HAMA, HAMA-5CPC, HAMA-10CPC, HAMA-20CPC. (D), (E), (F) and (G) Rheological and mechanical properties of t CPC, HAMA, HAMA-5CPC, HAMA-10CPC, HAMA-20CPC.


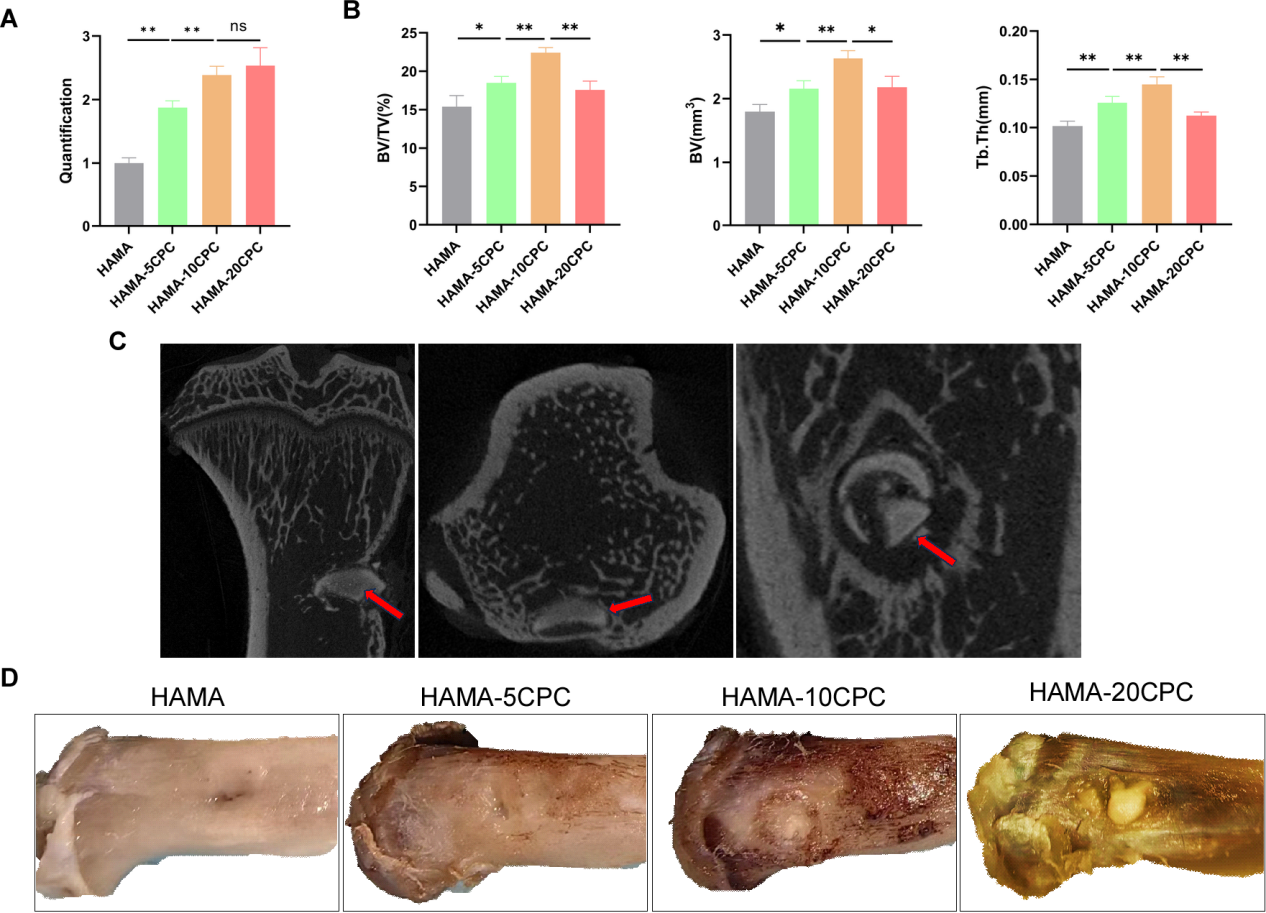


**Figure. S3.** (A) Alizarin red staining quantitative analysis. (B) Quantitative Analysis of Osteogenesis by microCT. (C) MicroCT images of tibial CPC residue after 4 weeks of material implantation, red arrow indicates the residual CPC. (D) Photograph of the tibia 4w after material implantation.


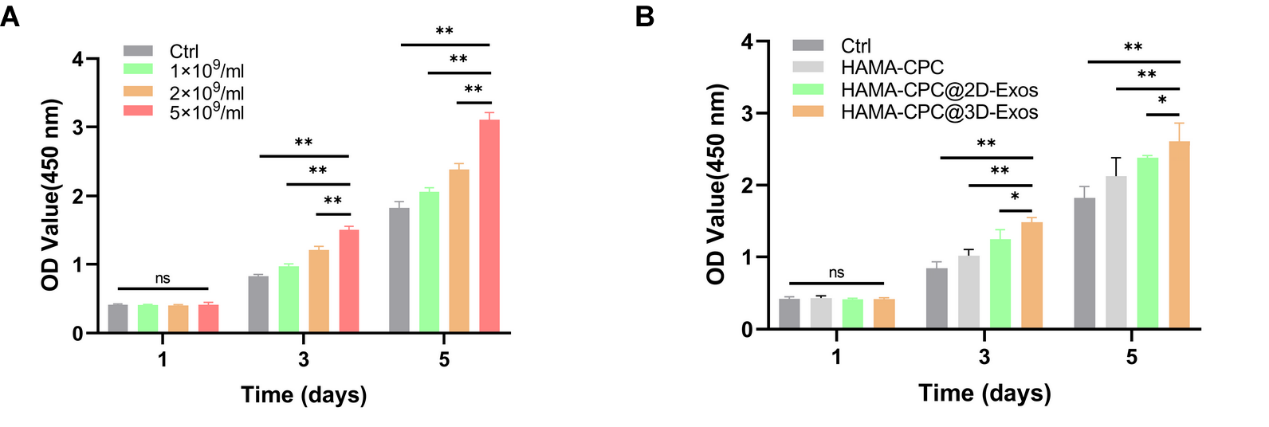


**Figure. S4.** (A) CCK-8 assay of BMSCs treated with varied concentrations of 3D-sEVs. (B) CCK-8 assay of BMSCs treated with HAMA-CPC@3D-sEVs.


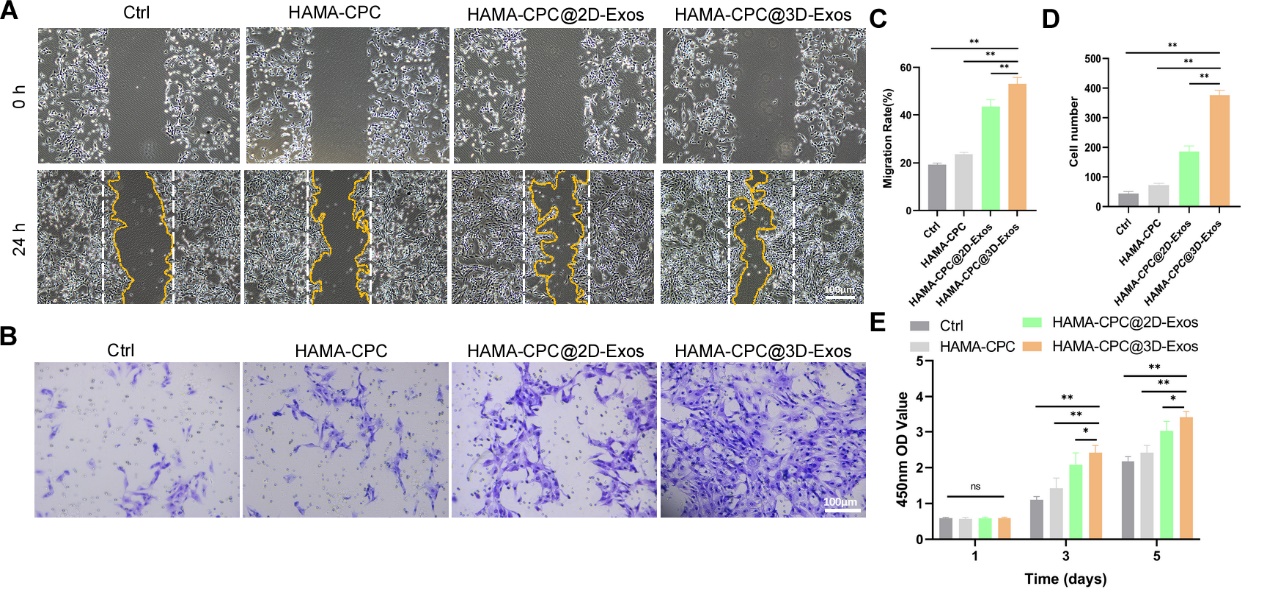


**Figure. S5**. (A) Wound healing assay of HUVECs at 24h and (C) quantitative analysis. (B) Transwell assay of HUVECs at 24h and (D) quantitative analysis. (E) CCK-8 assay of HUVECs.

**
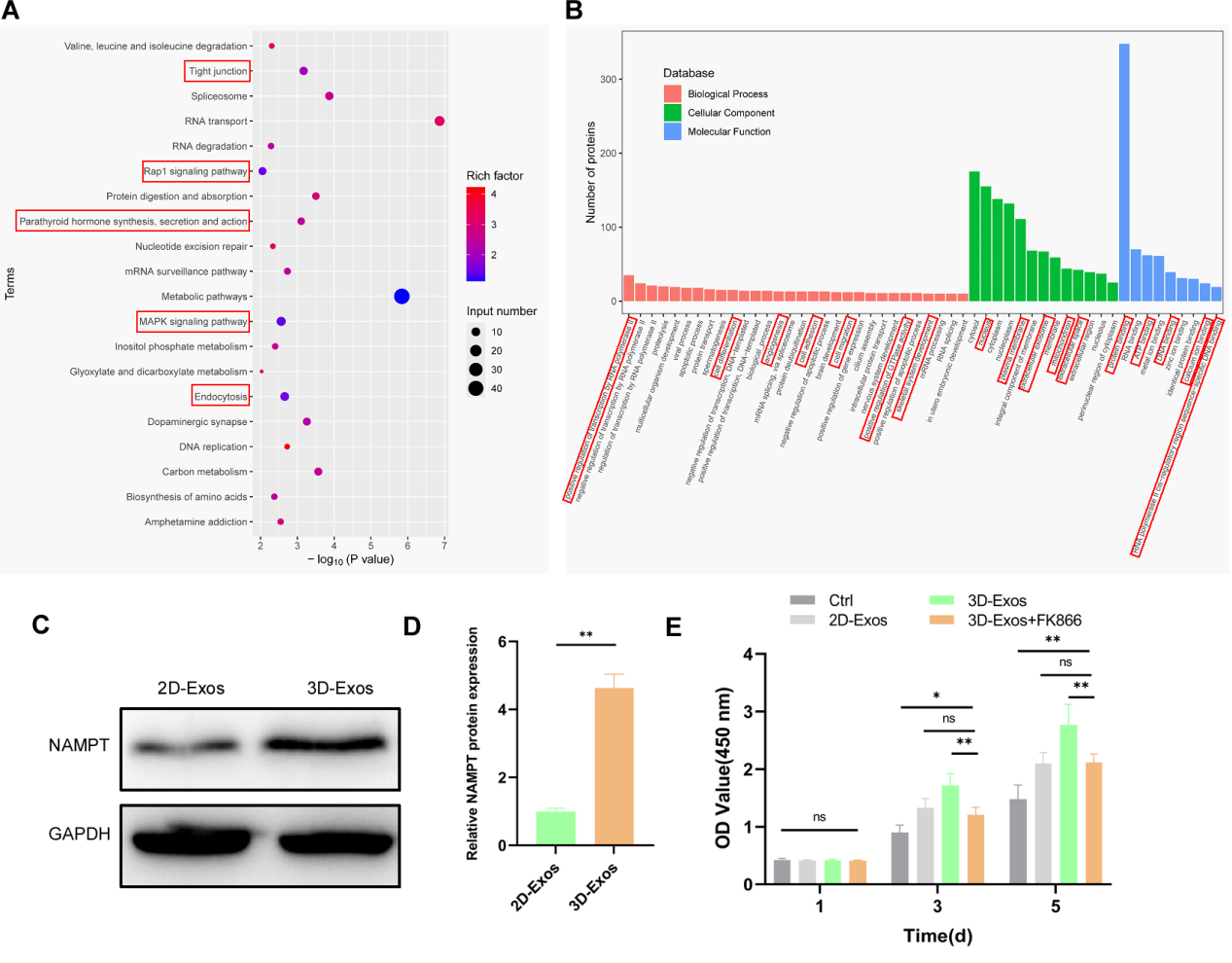
**

**Figure. S6.** (A) KEGG analysis of differentially expressed proteins highly expressed in 3D-sEVs. (B) GO enrichment analysis of differentially expressed proteins highly expressed in 3D-sEVs. (C) Western blot images and (D) quantitative analysis of NAMPT expression in 2D-sEVs and 3D-sEVs. (E) The effect of FK866 on the proliferation-promoting action of 3D-sEVs in HUVECs.

**
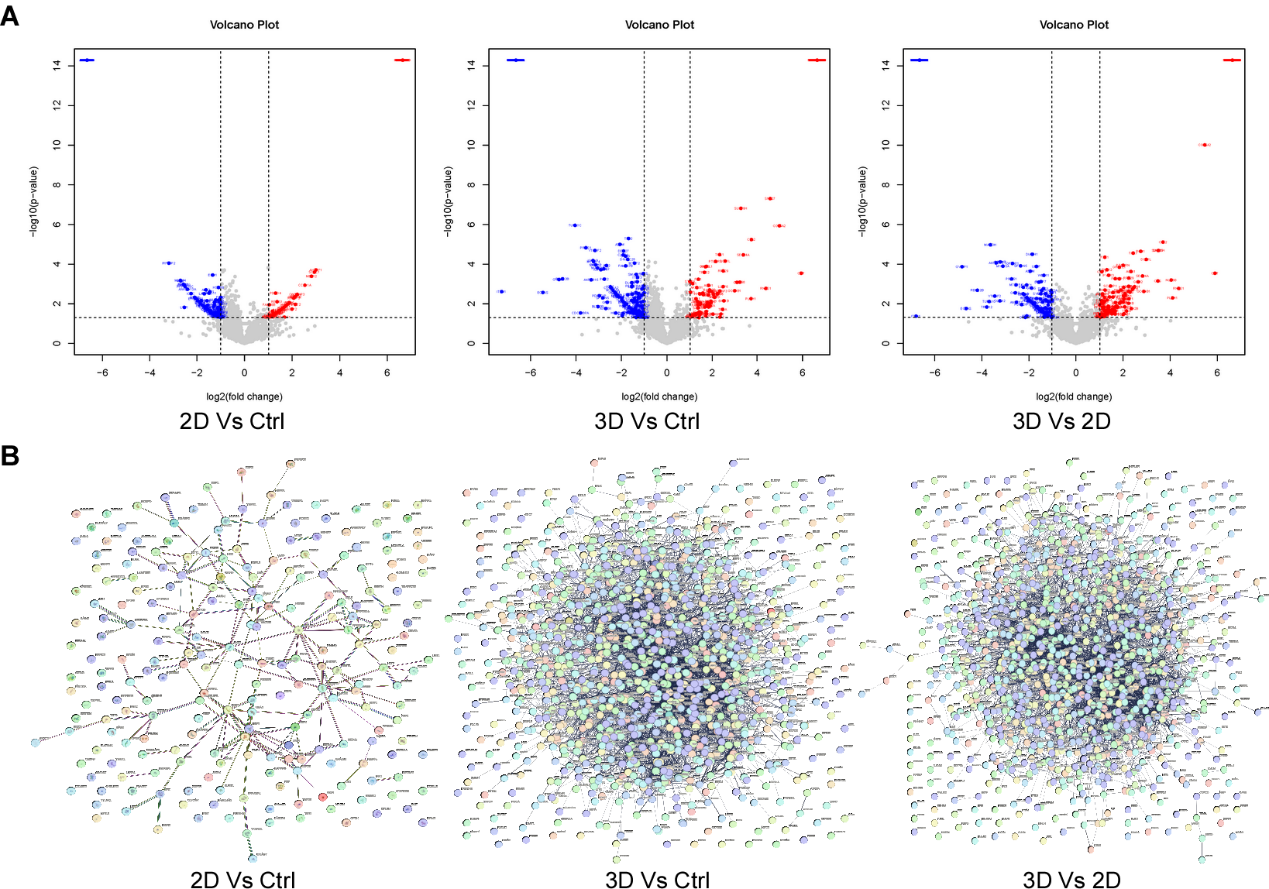
**

**Figure. S7.** (A) Volcano Plot of protein expression in HUVECs. (B) Protein-Protein interaction network in HUVECs.

**
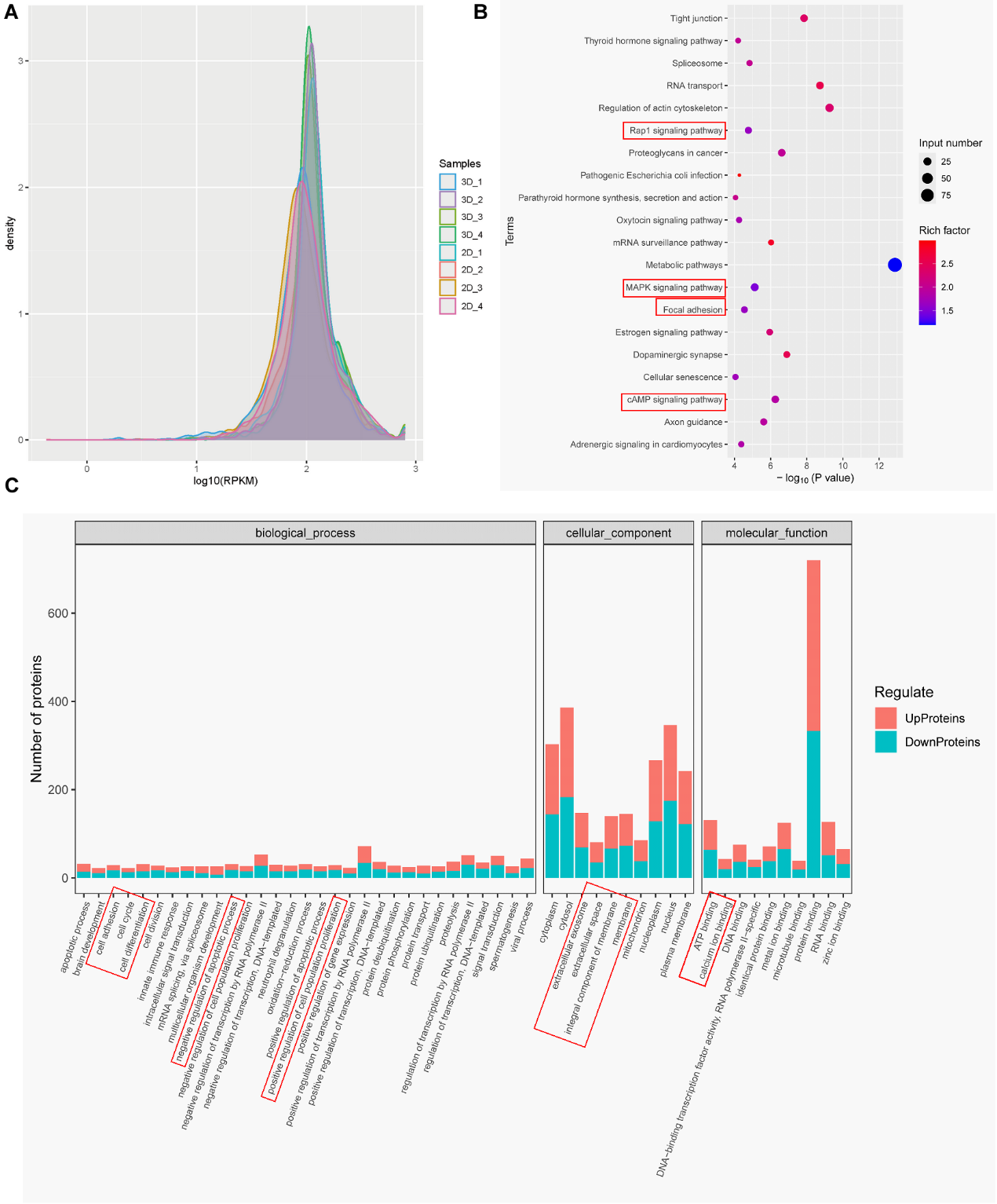
**

**Figure. S8.** (A) Protein abundance density distribution curve. (B) KEGG metabolic pathway map of differential proteins between 3D-sEVs and 2D-sEVs treated HUVECs. (C)GO enrichment chart for differential proteins between 3D-sEVs and 2D-sEVs treated HUVECs.

**
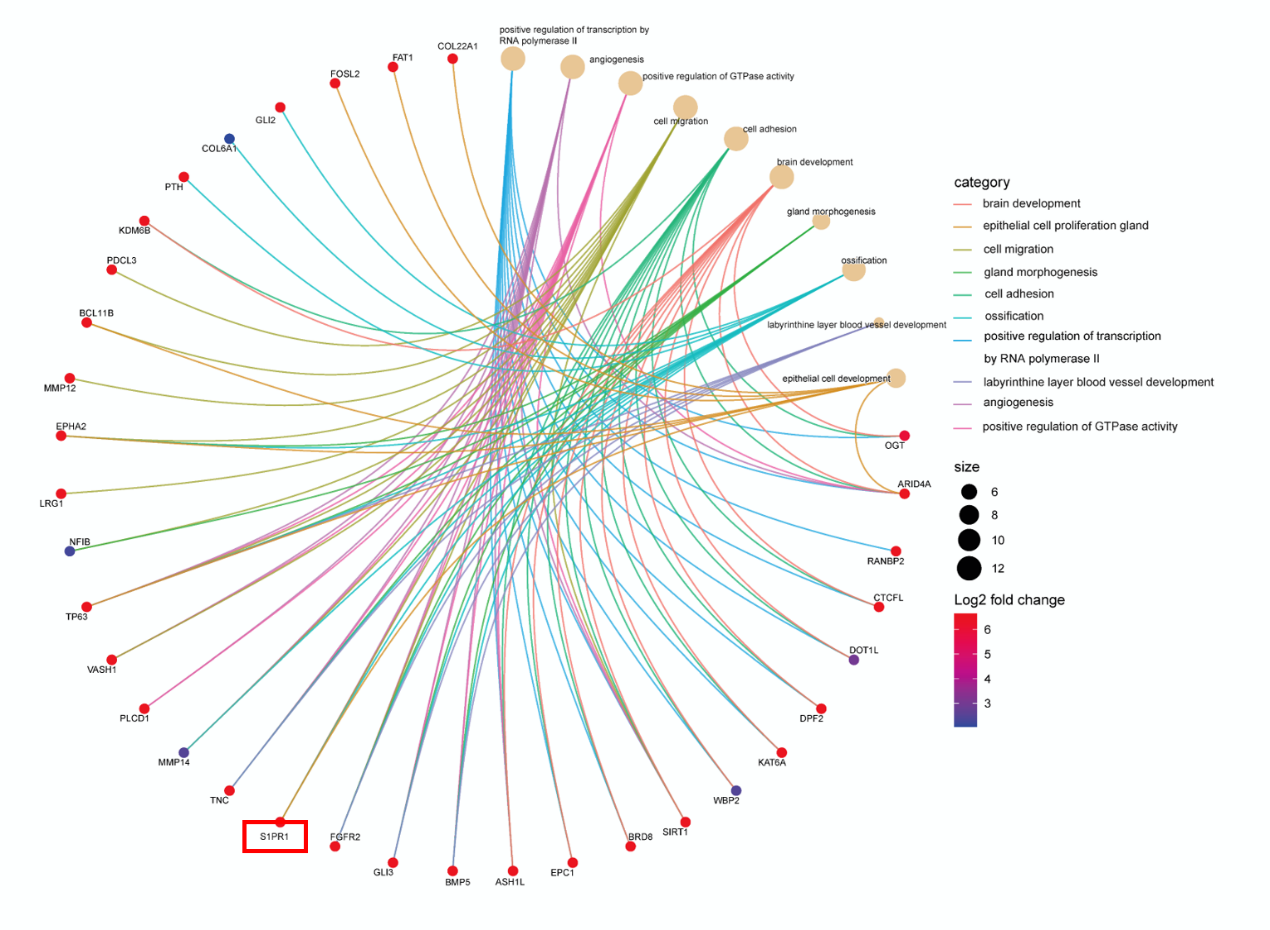
**

**Figure. S9.** Category network plot of differential proteins involved in biological processes such as angiogenesis, cell proliferation, migration, and adhesion.
